# Development and analysis of an *in vivo*-compatible metabolic network of *Mycobacterium tuberculosis*

**DOI:** 10.1186/1752-0509-4-160

**Published:** 2010-11-23

**Authors:** Xin Fang, Anders Wallqvist, Jaques Reifman

**Affiliations:** 1 Biotechnology HPC Software Applications Institute, Telemedicine and Advanced Technology Research Center, U.S. Army Medical Research and Materiel Command, Ft. Detrick, MD 21702, USA

## Abstract

**Background:**

During infection, *Mycobacterium tuberculosis *confronts a generally hostile and nutrient-poor *in vivo *host environment. Existing models and analyses of *M. tuberculosis *metabolic networks are able to reproduce experimentally measured cellular growth rates and identify genes required for growth in a range of different *in vitro *media. However, these models, under *in vitro *conditions, do not provide an adequate description of the metabolic processes required by the pathogen to infect and persist in a host.

**Results:**

To better account for the metabolic activity of *M. tuberculosis *in the host environment, we developed a set of procedures to systematically modify an existing *in vitro *metabolic network by enhancing the agreement between calculated and *in vivo-*measured gene essentiality data. After our modifications, the new *in vivo *network contained 663 genes, 838 metabolites, and 1,049 reactions and had a significantly increased sensitivity (0.81) in predicted gene essentiality than the *in vitro *network (0.31). We verified the modifications generated from the purely computational analysis through a review of the literature and found, for example, that, as the analysis suggested, lipids are used as the main source for carbon metabolism and oxygen must be available for the pathogen under *in vivo *conditions. Moreover, we used the developed *in vivo *network to predict the effects of double-gene deletions on *M. tuberculosis *growth in the host environment, explore metabolic adaptations to life in an acidic environment, highlight the importance of different enzymes in the tricarboxylic acid-cycle under different limiting nutrient conditions, investigate the effects of inhibiting multiple reactions, and look at the importance of both aerobic and anaerobic cellular respiration during infection.

**Conclusions:**

The network modifications we implemented suggest a distinctive set of metabolic conditions and requirements faced by *M. tuberculosis *during host infection compared with *in vitro *growth. Likewise, the double-gene deletion calculations highlight the importance of specific metabolic pathways used by the pathogen in the host environment. The newly constructed network provides a quantitative model to study the metabolism and associated drug targets of *M. tuberculosis *under *in vivo *conditions.

## Background

Tuberculosis (TB) continues to be a major health threat, with 9.2 million new cases and 1.7 million deaths reported worldwide in 2006 [1,2]. It has been estimated that one-third of the human population is infected with *Mycobacterium tuberculosis*, the causative agent of TB [3]. Worldwide efforts to treat and eliminate TB are confronting many obstacles, including drug-resistant bacterial strains, lack of compliance with the complicated drug regimens, and an increased patient population with compromised immune systems associated with acquired immunodeficiency syndrome [3,4].

In general, bacterial metabolism is an attractive drug target for two main reasons: *1*) metabolism is required for the bacterium to sustain itself and *2*) many bacterial metabolic targets are absent in humans. Novel efforts in developing drugs that target the intracellular metabolism of *M. tuberculosis *often focus on metabolic pathways that are specific to *M. tuberculosis *[5,6]. However, TB is a complex disease caused by bacterial populations located in discrete microenvironments of the host with access to a varying availability of nutrients [7]. This, coupled with the differences in bacterial metabolism under *in vivo *and *in vitro *conditions [8-10], creates a challenge in modeling and understanding the metabolic requirements of *M. tuberculosis *inside a host.

Recently, genome-scale metabolic network reconstructions for different organisms have enabled systematic analyses of metabolic functions and predictions of metabolism-related phenotypes [11,12]. By collecting all possible biochemical reactions for specific organisms, different groups have reconstructed metabolic networks for bacteria (e.g., for *Escherichia coli *[13], *Helicobacter pylori *[14], and *M. tuberculosis *[15,16]), eukaryotic microorganisms [17-19], mice [20], and even humans [21]. The Web site of the Systems Biology Research Group at the University of California, San Diego, provides a continuously updated list of genome-scale metabolic network reconstructions [22]. Analysis of metabolic networks can provide insights into an organism's ability to grow under specific conditions. For example, given a specific set of nutrient conditions, flux balance analysis (FBA) of metabolic networks can accurately predict microbial cellular growth rates [13,15-17,23]. In a recent work, Raghunathan et al. [24] used an approximate representation of in-host nutrient availability inferred from the literature to simulate the in-host metabolism of *Salmonella typhimurium*. Moreover, metabolic network analyses can then be used to identify organism-specific essential genes by predicting the attenuation of microbial growth of specific deletion mutants [13-17,19]. Metabolic genes that are essential for pathogen growth but are not present in humans constitute actual and potential drug targets [6,19].

Using the sequenced genome of *M. tuberculosis *[25] together with literature data on known metabolic reactions, extensive metabolic network reconstructions have been carried out for this organism [15,16]. Analyses of these networks based on FBA revealed that they contain sufficient information to predict growth rates and identify genes that are essential for the growth of *M. tuberculosis *in select media [15,16]. We have also used the *in vitro *network to model the drug-induced growth inhibition of *M. tuberculosis *when grown on defined media [26]. However, simulation of *M. tuberculosis *growth in an *in vivo *host environment based on an *in vitro *model is hindered by a lack of understanding of the pathogen's metabolism in the often poorly defined *in vivo *environment [7,27]. To experimentally explore the cellular activities of this pathogen in hosts, several methodologies have been developed. High-throughput gene expression experiments have been performed for *M. tuberculosis *in murine macrophage cells [28] and cells from mouse lung tissue [29]. Gene expression data have been interpreted, using the metabolic network of *M. tuberculosis*, to predict the production of mycolic acid [30]. Gene deletion experiments on *M. tuberculosis*, including individual [31-33] and high-throughput gene deletion studies [34], have identified genes that are essential in the murine host environment. In particular, Sassetti et al. developed the transposon site hybridization (TraSH) technique to identify genes required for *M. tuberculosis *growth in an *in vitro *medium [35] and genes specifically required for survival during *in vivo *infection [34]. This assay tested 2,979 genes, including a large fraction of genes known to be involved in metabolism.

For modeling, gene essentiality data *per se *are typically used to verify that a genome-scale metabolic network reconstruction is accurate; however, these data can also be used in the refinement process itself, either for specific pathways [36] or for the entire network [37-39], in which gene annotations, reactions, and biomass objective functions are adjusted based on gene essentiality data [37-39]. Moving away from these more or less *ad hoc *corrections, Kumar and Maranas [39] explored an automated and systematic way of reconciling *in silico*/*in vivo *growth predictions in large-scale metabolic networks. Here, we build on and extend these refinement methodologies to develop an enhanced set of systematic procedures to modify the *in vitro *metabolic network model of *M. tuberculosis iNJ*661 [15] and develop a network model that is more consistent with *in vivo *metabolism during the initial eight-week post-infection period. Importantly, the resultant network modifications provide indirect insights into the nutrient availability and metabolic states of *M. tuberculosis *in the mouse host environment. Furthermore, we used the newly developed *in vivo *network to predict the growth of double-deletion mutants to identify drug targets that are either specific for the host environment or common to both *in vivo *and *in vitro *conditions.

## Methods

To develop a *M. tuberculosis *metabolic network model commensurate with an *in vivo *cellular environment, we modified an existing network in two separate steps. First, we corrected an existing *in vitro *network model to account for missing or inconsistent chemical reactions and metabolites, and then, through a systematic set of procedures, we modified this network to be compatible with gene essentiality data generated under *in vivo *conditions.

### Modified *in vitro *network *iNJ*661m

We used the *iNJ*661 metabolic network model of *M. tuberculosis *H37Rv [15], which reproduces *in vitro *experimentally observed growth rates in different media, as the starting point for our work. Our modified *in vitro *network, *iNJ*661 m, models cellular growth in Middlebrook 7H9 medium supplemented with glucose and glycerol. We then used the GSMN-TB metabolic network of *M. tuberculosis *[16] to supplement reactions and metabolites in the modified network. We corrected the network with respect to biotin synthesis, fumarate and succinate synthesis, added the methylcitrate cycle, added a redundant annotation for the β-hydroxybutyryl-CoA dehydrogenase enzyme, and made minor changes to the biomass function (see Supplemental Section S1 in Additional file 1 for details). These modifications did not change the previously reported growth rates [15]. The resulting *iNJ*661m network model contained 663 genes, 838 metabolites, and 1,049 reactions. The developed network is provided in the Additional files in both Systems Biology Markup Language (Additional file 2) and Excel formats (Additional file 3).

### Prediction of essentiality of single genes and gene pairs

We used FBA of the metabolic networks to predict the essentiality of single genes. Using linear programming, FBA can maximize the cellular growth rate subject to the steady-state mass balance of all the intracellular metabolites and the stoichiometric constraints defined by the reactions [40-42]. For the metabolic network models, we performed FBA to calculate the growth rate of wild-type *M. tuberculosis *and the growth rates of all single-gene deletion mutants. If the ratio of a single-gene deletion mutant growth rate to wild-type growth rate was less than a threshold (0.2), we labeled the gene as essential; otherwise, it was deemed as non-essential. Since the growth rate ratios obtained from FBA were either <10^-4 ^or >0.2, the chosen threshold value of 0.2 differentiated growth rate ratios close to zero from those significantly higher than zero.

We compared the predicted gene essentiality with experimentally determined *in vivo *essentiality in mice [34,36] and defined four categories of predictions: true positives (TP), denoting genes that were predicted to be essential and were also essential in the experiment; true negatives (TN), denoting genes that were both predicted and experimentally determined to be non-essential; false negatives (FN), representing genes that were predicted to be non-essential but were experimentally essential; and false positives (FP), denoting genes that were predicted to be essential but were experimentally non-essential.

We predicted the synthetic essentiality of gene pairs in *M. tuberculosis *by calculating the growth rates of all possible double-gene deletion mutants in the metabolic network models. Two genes were classified as synthetically essential if each single individual gene deletion did not affect the growth of the organism, whereas the double-gene deletion impaired growth. Similar to the predictions for single-gene deletion mutants, we classified a deleted gene pair as synthetically essential if the ratio of the growth rate of the double-gene deletion mutant to that of the wild-type bacterium was smaller than the threshold (0.2) and the ratios for the two single-gene deletions were greater than the threshold.

### Modifications used to construct the *in vivo *network *iNJ*661v

**Figure 1:**
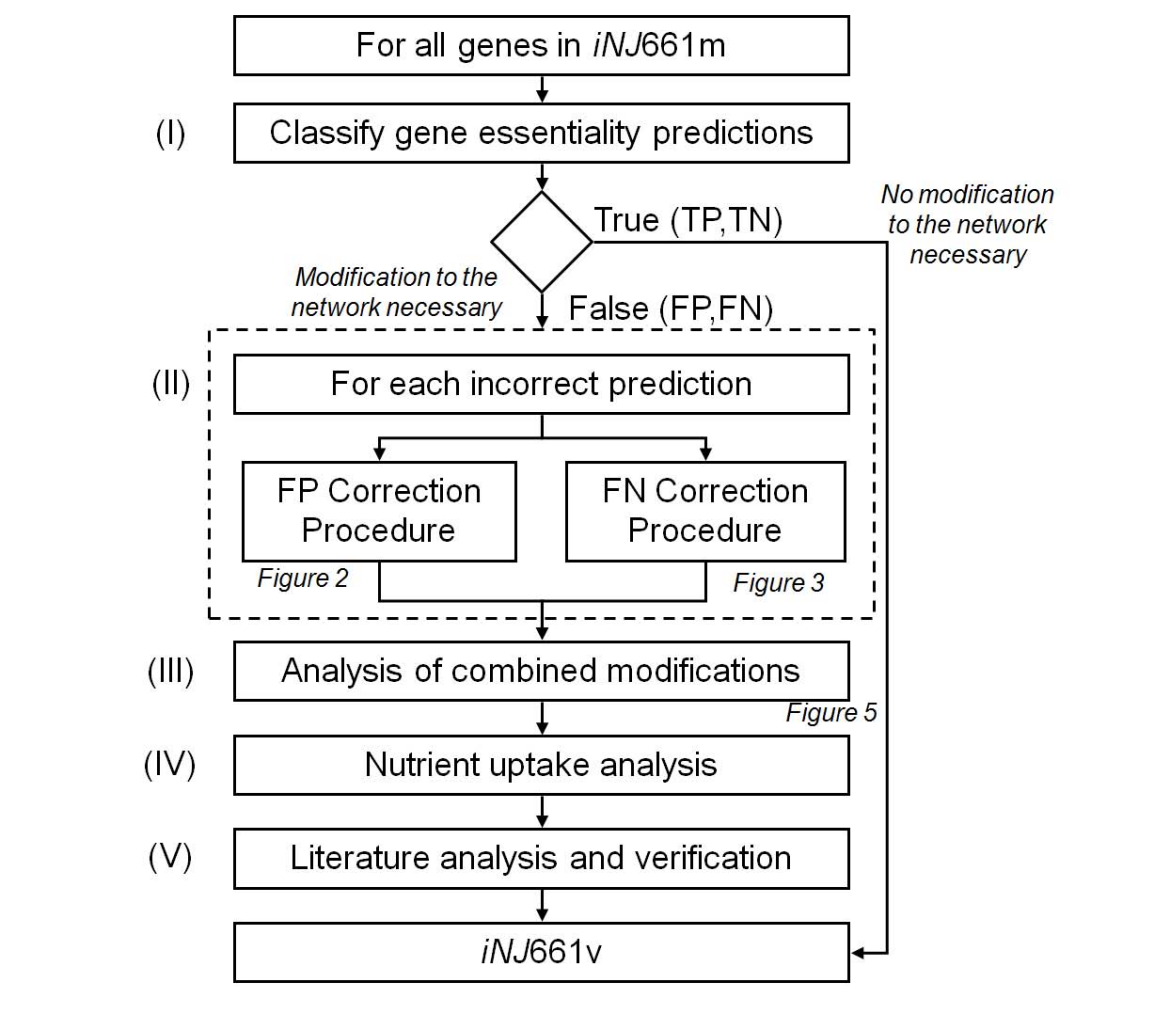
**Main steps for the development of the *iNJ*661v network**. In *Step I*, we compared the gene essentiality of *iNJ*661m with experimental *in vivo *data and identified the set of false positive (FP) and false negative (FN) predictions. In *Step II*, for each incorrect prediction, we attempted to obtain a set of possible modifications. In *Step III*, we combined all the suggested modifications for each different incorrect prediction and screened the network realizations to obtain adequate and consistent metabolic modifications. In *Step IV*, we analyzed the availability and blockage of nutrient uptakes. In *Step V*, we reviewed the relevant literature to verify the biochemical and biological veracity of the introduced modifications. TN, true negative; TP, true positive.

We developed a systematic set of procedures to optimally modify an existing metabolic network based on discrepancies in gene essentiality between computational predictions and experimental data. We applied the procedures to modify the original *in vitro *network, *iNJ*661 m, to optimally reproduce gene essentiality under an *in vivo *condition and create an *in vivo *network, *iNJ*661v. Figure 1 shows an overview of the five main steps of the procedure set, each of which is discussed in detail below. In *Step I*, we compared the gene essentiality of *iNJ*661m with experimental *in vivo *data and identified the set of FP and FN predictions. In *Step II*, for each incorrect prediction, we attempted to obtain a set of possible modifications to correct the predictions, commensurate with a minimum number of adjustments to *iNJ*661 m. In *Step III*, we combined all obtained modifications for each of the incorrect predictions and screened the sets of combined modifications to identify rational and consistent sets of metabolic modifications. In *Step IV*, we analyzed the availability and blockage of nutrient uptakes based on the original *iNJ*661m network and attempted to use small, chemically uncomplicated molecules as nutrient sources and to reduce the number of uptakes. Finally, in *Step V*, we reviewed the relevant literature to verify the biochemical and biological veracity of the introduced modifications. This set of procedure generated a number of different resultant networks, each representing a "minimal" adjustment that can optimally reproduce the given gene essentiality data. Because it is desirable to create a single unique network representation suitable for modeling and computational analysis, and because we do not have enough information to *a priori *discriminate against any particular network representation, we combined all networks as long as the combination did not generate any new incorrect predictions. Thus, the resultant network description *iNJ*661v corresponds to an unbiased assembly of minimal adjustments compatible with the experimental data.

#### Step I: Collation of the FP and FN predictions

The wrongly predicted FP and FN genes were collated as identified from the gene essentiality predictions described above.

#### Step II: FP correction procedure

**Figure 2:**
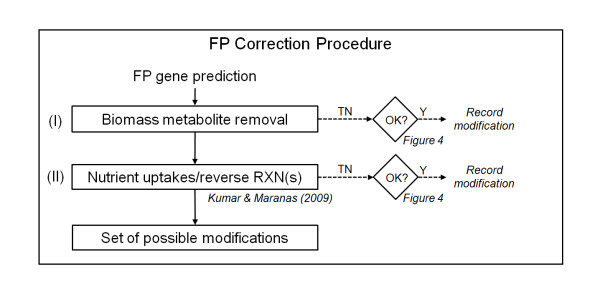
**Procedure to correct false positive (FP) gene essentiality predictions**. For each predicted FP gene, we attempted to correct the prediction by (*I*) removing metabolites from the biomass objective function and (*II*) introducing new nutrient uptakes and/or changing irreversible reactions to reversible (using the optimization model developed by Kumar and Maranas [39]). When a modification was successful, as determined by the criteria shown in Figure 4, we recorded and collected it in a set of possible modifications. TN, true negative; RXN, reaction; Y, yes.

Figure 2 shows the two types of network modifications we used to remove FP predictions. For each FP prediction, we first attempted to correct it by removing metabolites from the biomass objective function of the original *iNJ*661m network. In the original *iNJ*661 network, 16 vitamins and cofactors were included as part of the biomass based on *in vitro *gene essentiality data but without any experimental verification [15], suggesting that these metabolites might not be part of the biomass when *M. tuberculosis *grows in a different nutritional environment. Therefore, we systematically investigated the removal of one or more of these 16 metabolites. Initially, we removed one metabolite at a time from the biomass objective function and recorded the removals that corrected the FP prediction and caused no original TP prediction to become FN. If the single-metabolite removal did not correct the FP prediction, we expanded the removal to include all pairwise removals, and so on, until exhaustion or until the prediction was corrected. The second set of attempts to correct FP predictions introduced new nutrient uptakes and/or changed irreversible reactions to reversible. This procedure is based on the optimization model that Kumar and Maranas [39] developed to resolve FP inconsistencies in the *E. coli *metabolic network by adding a minimum number of reactions from a pool of reactions collected from multi-organism databases (MetaCyc [43] and KEGG [44]). We used the same optimization model, where the pool of potentially added reactions consisted of *1*) all uptake reactions blocked in the original *iNJ*661m network and *2*) all irreversible reactions in *iNJ*661m with their directions reversed. We used this model to obtain the modification(s) that changed the minimum number of irreversible reactions, and accepted the modification(s) if the changed irreversible reaction(s) was (were) reported as thermodynamically reversible in either the metabolic network of *E.coli *[13] or that of *Bacillus subtilis *[45]. These two networks include thermodynamic reversibility data. All attempts to correct for the FP predictions by removal of metabolites from the biomass objective function and addition of reactions were performed in parallel. Each modification used the same *iNJ*661m network as the starting point. The set of identified modifications was then analyzed together in *Step III: analysis of combined modifications*.

#### Step II: FN correction procedure

**Figure 3:**
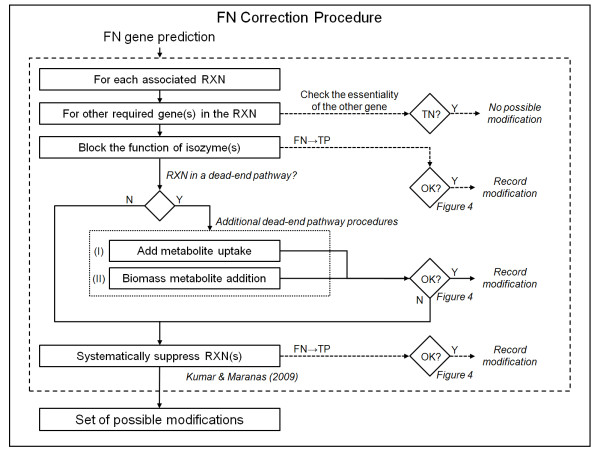
**Procedure to correct false negative (FN) gene essentiality predictions**. For each reaction associated with a FN gene, we first examined whether the reaction required the presence of both the FN gene and one or more TN genes. If this was not the case, we attempted to correct the FN prediction by blocking the functions of isozyme(s). Next, we examined whether the reaction was in a dead-end pathway, i.e., a pathway containing metabolites that cannot be produced, metabolites that cannot be consumed, or both. If this was the case, we added (*I*) metabolite uptakes or (*II*) metabolites to the biomass objective function. The last attempt was to correct the FN prediction by suppressing one or more reactions (using the optimization model developed by Kumar and Maranas [39]). When a modification was successful, as determined by the criteria shown in Figure 4, we recorded and collected it in a set of possible modifications. RXN, reaction; TN, true negative; TP, true positive; Y, yes; N, no.

Figure 3 shows the more complex procedures we used to correct the FN predictions. We initially listed all reactions catalyzed by the product of FN genes. For each reaction, we first examined whether the reaction required the presence of both a FN gene and one or more TN genes. If this were the case, no correction of the FN prediction was possible because a correction would have caused the predicted TN genes to become FP. If this were not the case, we attempted to correct the FN prediction by blocking the ability of isozyme(s) of the FN gene product to catalyze the reaction. Next, we examined whether the reaction was in a dead-end pathway, i.e., a pathway containing metabolites that cannot be produced, metabolites that cannot be consumed, or both. If a metabolite could not be produced, and the uptake reaction for this metabolite existed but was blocked in *iNJ*66 m, we restored the uptake. If a metabolite could not be consumed, we added this metabolite to the biomass objective function with a coefficient of 10^-6 ^mmol/gDW, that is, mmol per gram dry weight of *M. tuberculosis*. This coefficient was used in the biomass objective function of the original *iNJ*661 network to include metabolites for which quantitative biomass composition data were not available [15]. Finally, if the above analysis of the dead-end pathway did not correct the FN prediction or the reaction was not in a dead-end pathway, we attempted to correct the FN prediction by suppressing one or more reactions in *iNJ*661 m. For this procedure, we used the optimization model developed by Kumar and Maranas [39] for resolving FN inconsistencies and selected the modification(s) that suppressed the minimum number of reactions.

**Figure 4:**
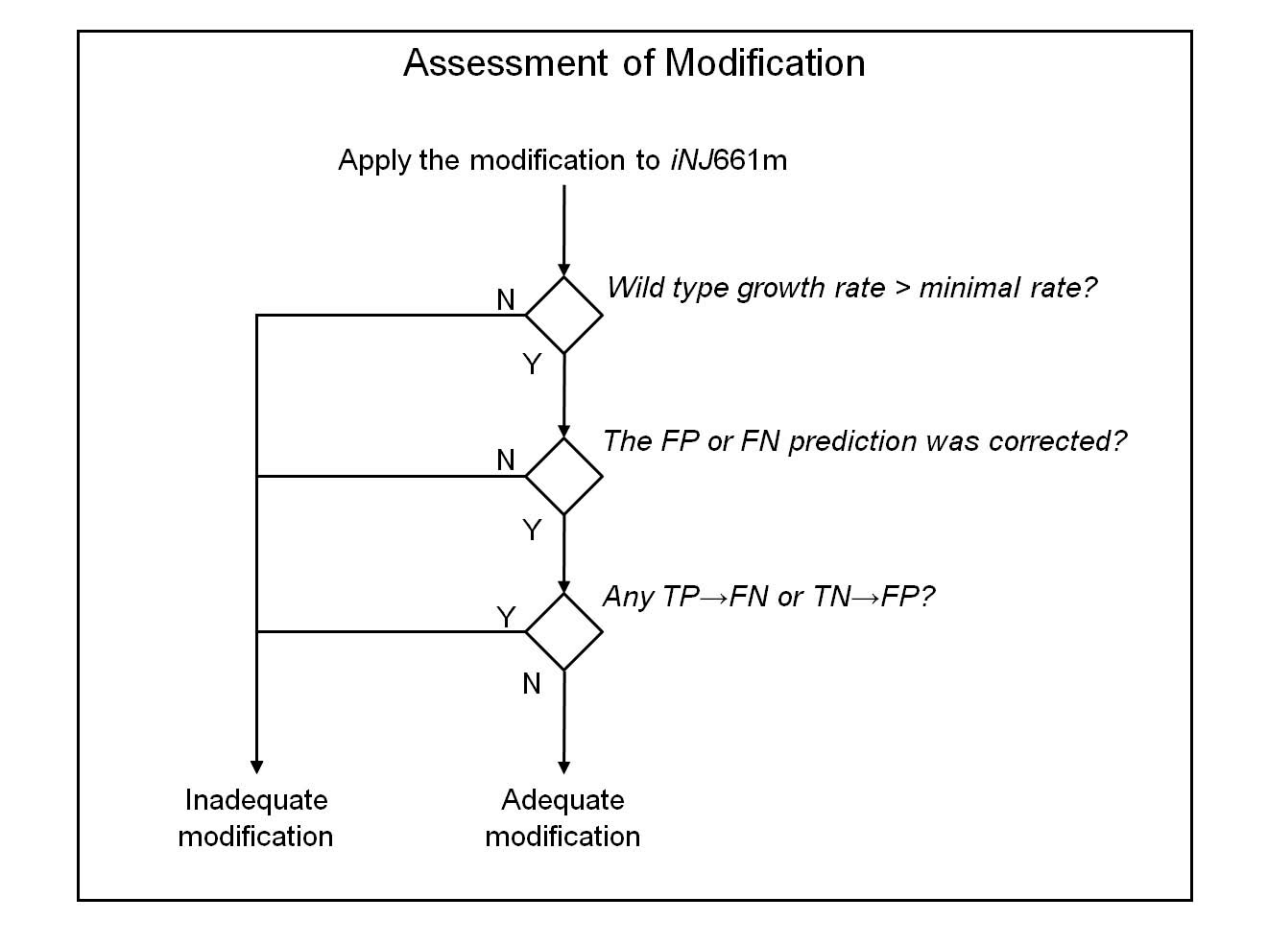
**Criteria to judge whether a modification for an incorrect prediction is adequate**. A modification was deemed to be adequate if, after applying the modification to *iNJ*661 m, the following criteria were met: *1*) the calculated wild-type growth rate was greater than the minimal rate (taken to be 0.027 h^-1^, according the growth rate of *M. tuberculosis *in mouse macrophages [32]), *2*) the false positive (FP) or false negative (FN) prediction was corrected, and *3*) no true positive (TP) or true negative (TN) prediction became FN or FP, respectively. Y, yes; N, no.

Note that, to correct for FP and FN predictions in the *Step II *procedures, we needed to assess whether a modification was adequate. Figure 4 shows the criteria we used to determine this. A modification was adequate if, after applying the modification to *iNJ*661 m, the following three criteria were satisfied: *1*) the calculated wild-type growth rate was greater than the minimal rate (taken to be 0.027 h^-1^, according to the growth rate of *M. tuberculosis *in mouse macrophages [32]), *2*) the FP or FN prediction was corrected, and *3*) no TP(TN) prediction became FN(FP).

#### Step III: Analysis of combined modifications

**Figure 5:**
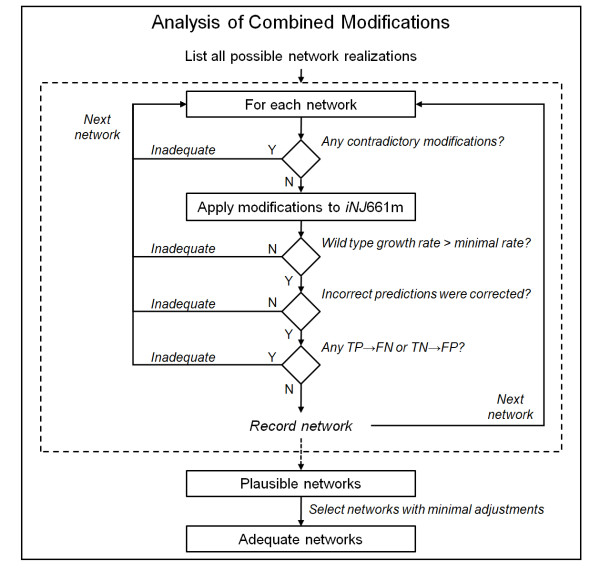
**Procedures to analyze combined modifications for different incorrect predictions**. Each network realization included one modification for every incorrect prediction. A plausible network realization satisfied the following criteria: *1*) the network contained no contradictory modification, and, after the combined modifications to *iNJ*661m were applied, *2*) the wild-type growth rate (calculated from flux balance analysis) was greater than the minimal rate (taken to be 0.027 h^-1 ^according the growth rate of *M. tuberculosis *in mouse macrophages [32]), *3*) incorrect predictions were corrected, and *4*) existing correct predictions before the applied modification were still correct. Finally, from all plausible network realizations, we selected the networks that were associated with minimum adjustment. TN, true negative; TP, true positive; FN, false negative; TN, true negative; Y, yes; N, no.

After the completion of *Step II*, there might be more than one modification used to correct for each of the FP or FN predictions, and we needed to analyze the consequences of combining these modifications into a single network description. Figure 5 summarizes these analyses. We first listed all possible network realizations, where each realization represented a set of combined modifications. Thus, if there were two possible modifications that correct for the prediction of *gene A*, three possible modifications that correct for the prediction of *gene B*, etc., then there would be 2*3*... possible network realizations, where each realization includes one modification for each incorrect prediction. Next, for each network realization, we checked whether the network contained contradictory modifications, e.g., one modification in the network blocked a reaction flux, whereas another modification allowed for the same reaction flux, or one modification added a metabolite to the biomass objective function, whereas another modification removed it. If contradictory modifications were not detected, then we applied the modifications to *iNJ*661m and verified that *1*) the wild-type growth rate (calculated from FBA) was greater than the minimal rate, *2*) incorrect predictions were corrected, and *3*) correct predictions (TP or TN) were still correct. If true, we recorded the network as plausible. Finally, from all plausible networks, we selected the ones that were associated with the smallest number of adjustments (i.e., the smallest number of irreversible reaction changes and reaction suppressions).

#### Step IV: Nutrient uptake analysis

In *Step II*, we allowed for the addition and removal of a fixed set of nutrient uptakes, leaving other nutrient uptakes (blocked or unblocked) of *iNJ*661m unexamined. Here, we re-examined these uptakes in light of the altered nutritional environment under *in vivo *conditions. The harsh nutritional environment that *M. tuberculosis *confronts when the bacterium infects a mouse suggests that nutrient uptake is limited and restricted to generally available small molecules as possible metabolite sources. Thus, we implemented a nutrient uptake-based analysis procedure to eliminate uptakes of complex nutrients as much as possible while minimizing the number of additional small-molecule uptakes and maintaining the compatibility with the *in vivo *gene essentiality data. The primary location of *M. tuberculosis *in a recently infected animal is the phagosomal compartments of macrophages. In this environment, large molecules are typically broken down into smaller ones. For example, macrophages decompose proteins into amino acids [46], which in turn are decomposed into smaller molecules, such as nitrite and nitrate [47]. Therefore, we assumed that the *in vivo *nutrient environment did not contain a significant abundance of all large molecules, such as amino acids, but rather was primarily composed of small molecules, such as nitrite, nitrate, ammonia, phosphate, and so on. However, as some large molecules may not be broken down, we still kept the uptakes for these molecules in two cases: *1) *if their uptakes was added during the correction of a false gene essentiality prediction (for example, fatty acids); and *2) *if deletions of these uptakes caused a growth rate lower than the defined threshold or generated new false gene essentiality predictions.

Before doing any calculation, we first assembled a set of available small-molecular-weight metabolites that *1*) contained the necessary atomic elements found among the biomass molecules, i.e., carbon, nitrogen, oxygen, phosphorus, sulphur, iron, potassium, and sodium; *2*) had a recorded uptake reaction in *iNJ*661m; and *3*) contained a minimum number of other elements. Based on the elements, these small-molecule metabolites were then grouped accordingly: carbon monoxide (CO) and carbon dioxide (CO_2_) for carbon; ammonium (NH_4_^+^), nitrite (NO_2_^-^), and nitrate (NO_3_^-^) for nitrogen; O_2 _for oxygen; phosphate (HPO_4_^2-^) for phosphorus; sulphate (SO_4_^2-^) for sulphur; ferrous (Fe^2+^) and ferric (Fe^3+^) ions for iron; K^+ ^for potassium; and Na^+ ^for sodium. Note that nitric oxide (NO) is a small nitrogen-containing molecule that was not included in the analysis as it is generated as part of the host defense system rather than acting as a nitrogen source [48]. Commensurately, we also blocked the reaction catalyzed by cytochrome *c *oxidase because experiments have suggested that NO prevents the function of this enzyme [48]. Note that this is the only literature source we used before proceeding to the literature analysis and verification in *Step V*.

For each network realization from *Step III*, we divided all uptake reactions into the following two sets: *1*) a "minimal" uptake set comprising the uptakes of H_2_O and H^+^, all uptakes added in *Step II*, and the uptakes of the small molecules defined above (minimal set); and *2*) an "extended" uptake set comprising all other defined uptake reactions in *iNJ*661m (extended set). Initially, we allowed uptake reactions from the minimal set and blocked all reactions from the extended set. As expected, this resulted in a non-biological network displaying an overall growth rate of zero. We then used the minimization procedure adopted from Kumar and Maranas [39] to determine the smallest set of uptake reactions in the extended set that restored a minimal growth rate and introduced no new FP or FN predictions.

Next, we fixed this set of uptake reactions and investigated which small-molecule uptake reactions from the minimal set were dispensable, i.e., we tried to find a minimal set of uptakes that was still compatible with growth. To do this, we allowed metabolite uptakes for all members of the small-molecule set and investigated removal of specific uptakes based on each element group as defined above. We did this by systematically removing, for each element group, all combinations of small-molecule uptakes and recording which combinations resulted in a wild-type growth rate that was greater than the minimal rate and where each of the TP and TN predictions were preserved. For example, for the carbon group, we investigated the possibilities of *1*) removing both the CO and CO_2 _uptakes, *2*) removing only the CO uptake, and *3*) removing only the CO_2 _uptake. When investigating the next element group, all other small-molecule metabolite uptakes were restored. Finally, we selected the minimal number of small-molecule uptakes for each element that was compatible with maintaining wild-type growth and preserving the true gene essentiality predictions.

All the computational correction procedures above (*Steps II-IV*) were fully automated and attempted to capture all feasible minimal modifications to the network that optimized compatibility with the gene essentiality data. Supplemental Section S2 in Additional file 1 shows a more detailed description of these procedures. Since all feasible corrections for each false prediction were collected, it was possible to obtain more than one resultant network. To generate the single *in vivo *network *iNJ*661v, we included all feasible modifications, as long as no new incorrect predictions or contradictory nutrient uptakes were generated.

#### Step V: Literature analysis and verification

We reviewed the available literature on *M. tuberculosis *to examine the biological rationale of our modifications. The presence of supporting data from the literature suggested that our systematic procedures could provide insights into *in vivo *metabolism. The absence of literature citations for specific modifications indicated the need for possible future experimental work to link metabolism and gene essentiality data.

### Statistic analyses of metabolic networks

We used statistical methods to compare the ability of the three metabolic network models (*iNJ*661, *iNJ*661 m, and *iNJ*661v) to predict experimental *in vivo *gene essentiality [34]. For each of the three networks, we obtained the total numbers of TP, TN, FN, and FP genes and compared the corresponding sensitivity and specificity. We also calculated Matthews correlation coefficients (MCCs) to evaluate the ability of the networks to predict and classify gene essentiality [49]. The MCC measures the correlation between observed and predicted binary classifications and ranges in values from +1 to -1, with +1 indicating a perfect prediction, 0 indicating a random prediction, and -1 indicating an inverse prediction. The calculated sensitivity, specificity, and MCCs depend on the value chosen for the growth rate ratio threshold to determine gene essentiality. To gauge the overall performance of the designed metabolic network model, we calculated receiver operating characteristic (ROC) curves for the studied networks. The ROC curve provides sensitivity as a function of 1 minus specificity across all possible thresholds [50], and, by estimating the 95% confidence interval of the area under the ROC curve (AUC), we can quantitatively assess and compare the global performance of each metabolic network model [51].

## Results

### Development of the modified *in vivo *network *iNJ*661v

Sassetti et al. [35] experimentally identified the genes essential for *M. tuberculosis *growth within an *in vitro *medium. We used the data from this *in vitro *experiment to verify the ability of *iNJ*661 and *iNJ*661m to predict *in vitro *gene essentiality. In addition, Sassetti and Rubin [34] adapted the TraSH technique to test for genes specifically required for the survival of *M. tuberculosis *during infection of the pathogen in mice [34]. Although this technique cannot positively identify *in vivo *essential genes whose deletion mutants are highly attenuated within the *in vitro *medium, the differentially identified set provides unique insights into the changed metabolic state of the pathogen [34]. In addition, Murphy et al. [36] showed that the *otsB2 *gene is essential for *M. tuberculosis *under *in vivo *conditions. We used the data from these two *in vivo *experiments [34,36] as the basis for our manipulations of metabolic reactions and metabolites to obtain a metabolic network (*iNJ*661v) that was compatible with pathogen growth under *in vivo *conditions.

Table 1 shows the predicted gene essentiality data based on FBA of *iNJ*661 [15], *iNJ*661 m, and GSMN-TB [16] as well as a comparison of the results with *in vitro *experimental datasets [35]. To verify our ability to correctly analyze the metabolic networks, we first repeated the original work of Jamshidi and Palsson [15] using the criterion that any gene whose deletion mutant had a growth rate less than that of the wild type, i.e., the mutant growth rate ratio was <1, was considered to be essential for growth. Our analysis of *iNJ*661 yielded 153 TP genes and 84 FN genes using a growth rate ratio threshold of 1.0. Due to minor numerical differences in cutoffs and constraint values in the FBA, these numbers were slightly different from the published results (154 TP and 83 FN) [15]. When the growth rate ratio threshold was lowered to 0.2, the sensitivity decreased from 0.65 to 0.57, whereas the specificity increased from 0.77 to 0.81. In addition, *iNJ*661 gave MCC values ranging from 0.39 to 0.42 depending on the specific threshold. We also performed the same calculations for *iNJ*661m and GSMN-TB. We obtained very similar results for *iNJ*661 m, suggesting that *iNJ*661m had the same ability to predict *in vitro *gene essentiality. The agreement between the GSMN-TB-predicted essentiality and the *in vitro *experimental data was slightly better than that for the other two networks, with MCC values for GSMN-TB ranging from 0.49-0.52.

**Table 1:** Comparison of predicted and experimental gene essentiality using different networks and different growth conditions.

Network	Experiment Condition	Threshold	Number of Gene Essentiality Predictions				Sensitivity	Specificity	Matthews Correlation Coefficient
						
			TP	FN	FP	TN			
*iNJ*661	*In vitro*	<1.0	153	84	71	236	0.65	0.77	0.42

*iNJ*661m	*In vitro*	<1.0	153	85	71	237	0.64	0.77	0.42

GSMN-TB	*In vitro*	<1.0	156	85	58	294	0.65	0.84	0.49

GSMN-TBv	*In vitro*	<1.0	160	81	75	277	0.66	0.79	0.45

*iNJ*661v	*In vitro*	<1.0	140	98	80	228	0.59	0.74	0.33

*iNJ*661	*In vitro*	≤0.2	135	102	59	248	0.57	0.81	0.39

*iNJ*661m	*In vitro*	≤0.2	135	103	59	249	0.57	0.81	0.39

GSMN-TB	*In vitro*	≤0.2	152	89	47	305	0.63	0.87	0.52

GSMN-TBv	*In vitro*	≤0.2	156	85	65	287	0.65	0.82	0.47

*iNJ*661v	*In vitro*	≤0.2	123	115	63	245	0.52	0.80	0.33

									

*iNJ*661	*In vivo*	<1.0	16	20	97	242	0.44	0.71	0.10

*iNJ*661m	*In vivo*	<1.0	16	20	94	246	0.44	0.72	0.11

GSMN-TB	*In vivo*	<1.0	10	34	76	317	0.23	0.81	0.03

GSMN-TBv	*In vivo*	<1.0	16	28	93	300	0.36	0.76	0.09

*iNJ*661v	*In vivo*	<1.0	31	5	77	263	0.86	0.77	0.41

*iNJ*661	*In vivo*	≤0.2	11	25	76	263	0.31	0.78	0.06

*iNJ*661m	*In vivo*	≤0.2	11	25	76	264	0.31	0.78	0.06

GSMN-TB	*In vivo*	≤0.2	10	34	65	328	0.23	0.83	0.05

GSMN-TBv	*In vivo*	≤0.2	16	28	83	310	0.36	0.79	0.11

*iNJ*661v	*In vivo*	≤0.2	29	7	52	288	0.81	0.85	0.47

A true positive (TP) prediction refers to a gene correctly predicted to be essential, whereas a false negative (FN) prediction refers to a gene incorrectly predicted to be non-essential. A false positive (FP) prediction refers to a gene incorrectly predicted to be essential, whereas a true negative (TN) prediction refers to a gene correctly predicted to be non-essential. Sensitivity = TP/(TP + FN). Specificity = TN/(TN + FP). Matthews correlation coefficient = (TP × TN - FP × FN)/[(TP + FP)(TP + FN)(TN + FP)(TN + FN)]^1/2^. GSMN-TBv indicates the GSMN-TB network with its *in vivo *biomass objective function.

In contrast to the *in vitro *results, the *in vivo-*predicted essentiality of *iNJ*661, *iNJ*661 m, and GSMN-TB was less satisfactory. Using the designated *in vivo *biomass composition formulation in the GSMN-TB network (indicated as GSMN-TBv) provided a slightly larger sensitivity in the essentiality prediction, but the improvement was only modest. The sensitivity for the predicted essentiality of these networks ranged from 0.23 to 0.44 depending on the threshold used. MCC values also decreased to 0.03-0.11 when we used these networks to predict *in vivo *essentiality. The relatively poor match between these predictions and the *in vivo *experimental data suggests that *iNJ*661, *iNJ*661 m, and GSMN-TB(v) are inappropriate to describe the metabolic activity of *M. tuberculosis *under *in vivo *conditions.

Therefore, we attempted to obtain the new network by modifying an existing metabolic network of *M. tuberculosis*. Among the two available networks [15,16], we selected *iNJ*661 as the starting point because it is based on the H37Rv strain of *M. tuberculosis *used in the *in vivo *gene essentiality experiments [34,36]. In contrast, the GSMN-TB construct is meant to be a general, non-strain specific model of *M*. *tuberculosis *metabolism. *iNJ*661 also successfully predicts the growth rate of *M. tuberculosis *H37Rv in two different media: Youmans and the "chemically defined rich culture media," while the growth rates predicted from GSMN-TB are only compared with experimental data for *M. bovis *BCG. Since the key aim of our work is to mimic as faithfully as possible the H37Rv strain, we choose not to use the GSMN-TB network as our starting point, although we used reactions relevant to the H37Rv strain from the GSMN-TB network to augment our construction.

We used *iNJ*661 m, the slightly improved version of *iNJ*661, as a starting point and performed the systematic procedures shown in Figure 1 to develop a modified metabolic network (*iNJ*661v) to better describe the *in vivo *metabolic activity of *M. tuberculosis*. Table 1 shows that the comparison between the *in vivo *experimental essentiality and the predicted essentiality of *iNJ*661m at the end of *Step I *yielded 76 FP and 25 FN predictions with a threshold of ≤0.2. All 25 FN and 76 FP genes were taken as input to *Step II*. The automated FP and FN correction procedures shown in Figures 2 and 3 were able to correct 24 of the 76 FP predictions and 18 of the 25 FN predictions, respectively. Additional file 1, Table S1 shows all the possible minimal corrective modifications for each of 42 (24 + 18) predictions.

Since there were multiple ways in which we could combine the different modifications, it became necessary to try to reduce the number of possible network realizations, as outlined in *Step III*. As shown in Additional file 1, Table S1, we had 35 groups of genes whose products each catalyze the same reaction and whose predictions can be corrected by creating the necessary conditions that makes the reaction essential for FN predictions or non-essential for FP predictions. For 30 groups there is only one possible modification, for two groups there are five possible modifications each, and for three groups there are two possible modifications each, resulting in a total of 1^30 ^× 5^2 ^× 2^3 ^= 200 possible network realizations. Next, we examined each one of these using the criteria shown in Figure 5 to weed out inadequate networks. Table 2 shows the modifications that survived this analysis, resulting in 31 groups with only one possible modification and four groups with two possible modifications each, resulting in a total of 1^31 ^× 2^4 ^= 16 plausible network realizations.

**Table 2:** Summary of modifications to correct gene essentiality predictions after Step III.

**Gene Group No**.	**Gene No**.	Gene Locus	Gene Name	FP/FN	Pathway	Function/Reaction	Modification to Correct the False Gene Essentiality Prediction	Supporting Literature (References)
1	1	*Rv1099c*	*Rv1099c*	FN	Glycolysis/gluconeogenesis	Convert fructose-1,6-bisphosphate into fructose-6-phosphate	(1) Blocked the uptake of glucose from environment	[8,28,32,52-57]

2	2	*Rv2702*	*ppgK*	FN	Glycolysis/gluconeogenesis	Conversion between glucose-6-phosphate and glucose	(1) Blocked the conversion from maltose to glucose and blocked the uptake of glucose (2) Blocked the conversion between maltose and trehalose and blocked the uptake of glucose	[8,28,32,52-57]

3	3 4 5	*Rv1350* *Rv1483* *Rv2947c*	*fabG2* *inhA* *pks15*	FP FP FP	Fatty acid metabolism	Synthesis of fatty acids	(1) Allowed the uptakes of the following fatty acids: hexadecanoate, octadecanoate, octanoate, dodecanoate, arachidic acid, and hexacosanoate	[8,28,32,52-57]

4	6	*Rv2503c*	*scoB*	FP	Fatty acid metabolism	Functions as 3-oxoacid CoA-transferase	(1) Let the reaction catalyzed by acetyl-CoA:acetoacetyl-CoA transferase be reversible	

5	7	*Rv3229c*	*desA3*	FN	Fatty acid metabolism	Palmitoyl-CoA desaturation	(1) Blocked the synthesis of hexadecenoate	[8,28,32,52-57]

6	8	*Rv1185c*	*fadD21*	FN	Fatty acid metabolism	Synthesis of fatty acid-CoA	(1) Blocked the ability of *fadD9 *(*Rv2590*), *fadD24 *(*Rv1529*), and *fadD23 *(*Rv3826*) to catalyze the synthesis of fatty acid-CoA	

7	9	*Rv0098*	*Rv0098*	FN	Fatty acid metabolism	Mycolic acid synthesis	(1) Blocked the ability of *fabG1 *(*Rv1483*) to catalyze the same reaction	

8	10	*Rv2483c*	*plsC*	FN	Fatty acid metabolism	Synthesis of 1,2-diacyl-*sn*-glycerol 3-phosphate (a phospholipid)	(1) Blocked the ability of *Rv2182c *to catalyze the same reaction	

9	11	*Rv1416*	*ribH*	FP	Vitamin and cofactor metabolism	Synthesis of riboflavin	(1) Removed riboflavin and flavin mononucleotide (FMN) from the biomass objective function	

10	12	*Rv1412*	*ribC*	FP	Vitamin and cofactor metabolism	Synthesis of riboflavin	(1) Removed riboflavin and FMN from the biomass objective function	

11	13	*Rv2671*	*ribD*	FP	Vitamin and cofactor metabolism	Synthesis of riboflavin precursor	(1) Removed riboflavin and FMN from the biomass objective function	

12	14	*Rv2786c*	*ribF*	FP	Vitamin and cofactor metabolism	Synthesis of FMN from riboflavin	(1) Removed FMN from the biomass objective function	

13	15	*Rv2421c*	*Rv2421c*	FP	Vitamin and cofactor metabolism	Synthesis of deamino-NAD^+^	(1) Removed nicotinamide adenine dinucleotide (NAD) and nicotinamide adenine dinucleotide phosphate (NADP) from the biomass objective function	

14	16	*Rv1596*	*nadC*	FP	Vitamin and cofactor metabolism	Functions as nicotinate-nucleotide diphosphorylase	(1) Removed NAD and NADP from the biomass objective function	

15	17	*Rv3215*	*entC*	FP	Vitamin and cofactor metabolism	Synthesis of isochorismate	(1) Removed menaquinol 8 from the biomass objective fucntion	

16	18	*Rv1568*	*bioA*	FN	Vitamin and cofactor metabolism	Synthesis of a precursor of biotin	(1) Added biotinyl-5'-AMP to the biomass objective function	

17	19	*Rv1569*	*bioF*	FN	Vitamin and cofactor metabolism	Synthesis of a precursor of biotin	(1) Added biotinyl-5'-AMP to the biomass objective function and blocked the ability of *bioF2 *(*Rv0032*) to catalyze the same reaction	

18	20	*Rv1589*	*bioB*	FN	Vitamin and cofactor metabolism	Synthesis of biotin	(1) Added biotinyl-5'-AMP to the biomass objective function	

19	21	*Rv2211c*	*gcvT*	FN	Vitamin and cofactor metabolism	Conversion between 5-formyltetrahydrofolate and 5,10-methenyltetrahydrofolate	(1) Added the metabolite 5-formyltetrahydrofolate to the biomass objective function	

20	22	*Rv3001c*	*ilvC*	FP	Amino acid metabolism	Synthesis of 2,3-dihydroxy-3-methylbutanoate and 2,3-dihydroxy-3-methylpentanoate	(1) Allowed the uptakes of isoleucine and valine	[61]

21	23	*Rv3002c*	*ilvN*	FP	Amino acid metabolism	Synthesis of acetolactate	(1) Allowed the uptake of valine	[61]

22	24 25 26 27	*Rv2220c* *Rv1878* *Rv2222c* *Rv2860c*	*glnA1* *glnA3* *glnA2* *glnA4*	FP FP FP FP	Amino acid metabolism	Synthesis of glutamine	(1) Let the reaction of glutamate synthesis from glutamine to be reversible (2) Let the conversion to CTP and glutamate from UTP and glutamine be reversible	

23	28	*Rv3754*	*tyrA*	FP	Amino acid metabolism	Functions as prephenate dehydrogenase	(1) Allowed the uptake of tyrosine	

24	29	*Rv3042c*	*serB2*	FN	Amino acid metabolism	Remove a phosphate group from phosphoserine to produce serine	(1) Blocked the ability of *serB *(*Rv0505c*) to catalyze the same reaction	

25	30	*Rv2231c*	*cobC*	FN	Amino acid metabolism	Convert glutamate into histidinol-phosphate	(1) Blocked the ability of *hisC2 *(*Rv3772*) and *hisC *(*Rv1600*) to catalyze the conversion	

26	31	*Rv2945c*	*lppX*	FN	Transport	Transport phthiocerol dimycocerosate A and phenol phthiocerol dimycocerosate out of the cell	(1) Added extracellular phthiocerol dimycocerosate A to the biomass objective function (2) Added extracellular phenol phthiocerol dimycocerosate to the biomass objective function	[62]

27	32 33 34	*Rv1236* *Rv1237* *Rv1238*	*sugA* *sugB* *sugC*	FN	Transport	Transport of glucose, maltoheptaose, maltose, ribose, trehalose, and xylose into cell	(1) Allowed xylose uptake and added xylose to the biomass objective function	

28	35	*Rv3236c*	*kefB*	FN	Transport	Transport of K^+ ^and Na^+ ^into the cell	(1) Blocked the function of potassium ABC transporter (2) Blocked the function of the Na^+ ^antiporter	

29	36	*Rv1699*	*pyrG*	FP	Nucleotide metabolism	Synthesis of CTP from UTP	(1) Allowed the uptake of cytidine	

30	37	*Rv1385*	*pyrF*	FP	Nucleotide metabolism	Functions as orotidine-5'-phosphate decarboxylase	(1) Allowed the uptake of cytidine	

31	38	*Rv2139*	*pyrD*	FP	Nucleotide metabolism	Functions as dihydroorotic acid dehydrogenase	(1) Allowed the uptake of cytidine	

32	39	*Rv3393*	*iunH*	FP	Nucleotide metabolism	Hydrolysis of inosine	(1) Let the reaction catalyzed by ribokinase be reversible	

33	40	*Rv2465c*	*rpi*	FP	Pentose phosphate pathway	Functions as ribose-5-phosphate isomerase	(1) Allowed the secretion of D-arabinose	

34	41	*Rv3628*	*ppa*	FP	Multiple pathways	Functions as inorganic diphosphatase	(1) let the reaction catalyzed by nucleoside triphosphate tripolyhydrolase of deoxy-GTP (dGTP) to be reversible	

35	42	*Rv3588c*	*Rv3588c*	FN	Multiple pathways	Conversion between carboxylic acid and carbon dioxide	(1) Blocked the ability of *Rv3273 *to catalyze the same reaction	

Of the 25 genes that were incorrectly predicted to be non-essential (FN) under *in vivo *conditions, 18 genes were corrected and became essential in the *iNJ*661v network. Of the 76 genes that were incorrectly predicted to be essential (FP) under *in vivo *conditions, 24 genes were corrected and became non-essential in the *iNJ*661v network. We classified the overall 42 (18 + 24) genes into 35 gene groups, defined as a group of genes whose products catalyze the same reaction(s).

In *Step IV*, we re-examined the assigned nutrient uptakes in the networks as outlined in the Methods Section. This analysis was performed for each of the 16 network realizations from *Step III*, each yielding the same set of uptakes. Table 3 shows that this set of uptakes comprises the default uptakes of H_2_O and H^+ ^(these two metabolites are always considered to be available), a minimum number of uptakes of small molecules, uptakes added based on the *Step II *analysis, and glycerol. We further decreased the upper limit of glycerol input to 0.06 mmol·h^-1^·gram dry weight^-1 ^until lower values of the glycerol input caused the *fum *gene to be incorrectly predicted as essential under *in vivo *conditions. Each of the resultant 16 networks contained the same set of uptakes and yielded the same number of correct predictions. Since we had no prior information to discriminate among these networks, we applied all modifications to *iNJ*661 m. Because this combination did not generate any new incorrect gene essentiality predictions, we designated this network as the "optimal" unbiased construction of the *in vivo *network *iNJ*661v.

**Table 3:** Nutrient uptakes in the iNJ661v network.

Uptake type		Nutrients	Supporting Literature (References)
Default uptakes		H_2_O H^+^	

	Oxygen	O_2_	[7,31]
	
	Phosphorus	HPO_4_^2-^	
	
Uptakes of small	Sulphur	SO_4_^2-^	
	
molecules	Iron	Fe^3+^	[64]
	
	Potassium	K^+^	
	
	Sodium	Na^+^	
	
	Nitrogen	NO_3_^-^, NH_4_^+^	[63]

Uptakes added in *Step II*		Hexadecanoate, octadecanoate, octanoate, dodecanoate, arachidic acid, and hexacosanoate	[8,28,32,52-57]
		
		Isoleucine and valine	[61]
		
		cytidine and xylose	

Other necessary uptakes		Glycerol	[8,28,32,52-57]

The *iNJ*661v network contained 663 genes, 838 metabolites, and 1,049 reactions and is provided in the Additional files in both Systems Biology Markup Language (Additional file 4) and Excel formats (Additional file 5). Based on this network, we re-calculated gene essentiality through FBA and compared the predictions with the experimental essentiality data in mice [34,36]. Table 1 shows the results from the comparison of the *in vivo *gene essentiality predictions of *iNJ*661v with those obtained using the *in vitro *networks. The sensitivity and specificity of the *iNJ*661v network model were substantially larger than those of the other two networks regardless of the threshold used to determine essentiality. When *iNJ*661v was used to predict *in vivo *essentiality, we gained in our ability to classify essentiality (MCC values of ~0.41-0.47) compared with using the *in vitro *networks (MCC values of ~0.06-0.11). Thus, a significant correlation between experimental gene essentiality in mice and calculated mutant growth rates was only present in *iNJ*661v.

**Figure 6:**
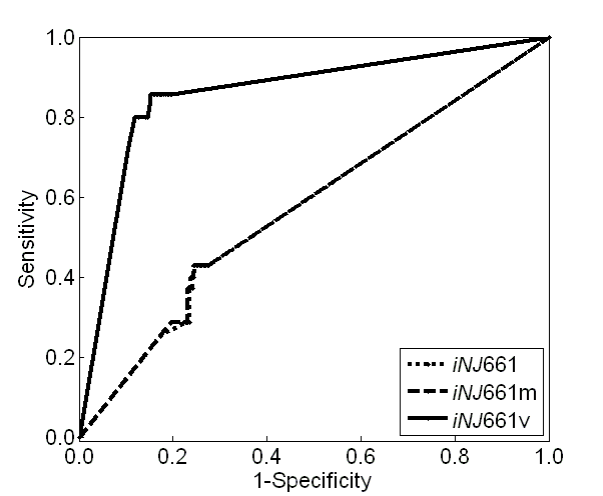
**Receiver operating characteristic (ROC) curves for gene essentiality predictions of *Mycobacterium tuberculosis***. Sensitivity [TP/(TP + FN)] and 1 minus specificity [1 - TN/(TN + FP)] (where TP: true positive, FN: false negative, TN: true negative, and FP: false positive) were calculated as a function of the growth ratio thresholds used to determine gene essentiality in three different network models: *iNJ*661 (dotted curve), *iNJ*661m (dashed curve), and *iNJ*661v (solid curve).

We further applied threshold-independent statistical tests to compare the abilities of *iNJ*661, *iNJ*661 m, and *iNJ*661v to predict experimental gene essentiality under *in vivo *conditions. Figure 6 shows the ROC curves of the three networks. For each curve, we obtained the 95% confidence interval of the AUC (*iNJ*661: 0.57 ± 0.09, *iNJ*661m: 0.57 ± 0.09, and *iNJ*661v: 0.84 ± 0.06). The AUCs of the *in vitro *models *iNJ*661m and *iNJ*661 were not significantly larger than those of random predictions (0.50), indicating the inability of these networks to predict *in vivo *essentiality. Conversely, the AUC of *iNJ*661v was significantly larger than those of the other two networks, demonstrating that *iNJ*661v was better able to predict experimental gene essentiality in mice.

After *Step IV*, the *iNJ*661v network still contained several incorrect gene essentiality predictions. Table 4 shows the seven FN predictions that we were unable to correct. We failed to correct the predictions for the *atpB*, *nirA*, *proV*, *accD1*, and *cobL *genes because each one of them is required together with one or more TN genes. For example, the FN gene *proV *is required together with the TN genes *proW*, *proX*, and *proZ *for the transport of choline, carnitine, glycine betaine, and proline into the cell [15]. Any "correction" of *proV *would change the TN predictions for *proW*, *proX*, and *proZ *into FP. It is likely that the gene products of *proV *have other essential functions that may or may not be related to metabolic functions and were not accounted for in the current network description. Conversely, we failed to correct the prediction for the *Rv3534c *gene because it belongs to a pathway containing a metabolite without a source. Thus, further studies are necessary to discover how this metabolite is synthesized and how this information can be incorporated into the network.

**Table 4:** False negative (FN) predictions that could not be corrected by our network modifications.

Gene Locus	Gene Name	Pathway	Function/Reaction	Reasons Why Network Modification Could Not Be Made
*Rv1304*	*atpB*	Purine metabolism	Synthesis of ATP	The product of this gene catalyzes the reaction together with that of the TN gene *atpH*

*Rv2391*	*nirA*	Vitamin and cofactor metabolism	Reduction of nitrite	The product of this gene catalyzes the reaction together with that of the TN gene *nirB*

*Rv3758c*	*proV*	Transport	Transport of choline, L-carnitine, glycine betaine, and proline	The product of this gene catalyzes the transport of choline, L-carnitine, glycine betaine, and proline together with the TN genes *proZ*, *proW*, and *proX*

*Rv2502c*	*accD1*	Sugar metabolism	Convert propionyl-CoA into *S*-methylmalonyl-CoA	The product of this gene catalyzes the reaction together with the TN gene *accA2*

*Rv2072c*	*cobL*	Porphyrin metabolism	Production of *S*-adenosyl-L-homocysteine	The product of this gene catalyzes the synthesis of cobalamin together with the TN genes *cobK, cobM*, *cobH*, *cobN*, *cobI*, and *cobG*

*Rv3534c*	*Rv3534c*	Pyruvate metabolism	Convert 4-hydroxy-2-oxopentanoate into pyruvate	In a pathway without any synthesis or uptake reaction for the metabolite 4-hydroxy-2-oxopentanoate

*Rv2241*	*aceE*	Glycolysis/gluconeogenesis	Convert pyruvate into acetyl-CoA	Unable to determine the reason

A false negative (FN) prediction refers to a gene incorrectly predicted to be non-essential.

### Literature Analysis and Verification

By design, our automated and systematic analysis of the *in silico*/*in vivo *growth inconsistencies generated by the original *iNJ*661m metabolic network created a network (*iNJ*661v) that was more compatible with *in vivo *growth. The metabolic modifications reflected how the pathogen adjusts its metabolism to adapt to the environment that *M. tuberculosis *confronts during infection in the mouse. Here, we discuss the performed modifications vis-a-vis the available relevant literature within the context of the affected metabolic pathways. Table 2 shows the reviewed literature associated with each of the performed modifications ordered by the affected pathway.

In the "glycolysis/gluconeogenesis" pathways, we deleted the uptake of glucose from the environment and blocked the synthesis of glucose from maltose and trehalose to correct the FN predictions of the *Rv1099c *and *ppgK *genes. This modification suggested that the host environment may lack glucose and, thus, forces *M. tuberculosis *to generate glucose through gluconeogenesis (the pathway to synthesize glucose from the citric acid cycle). In the "fatty acid metabolism" pathways, to correct the FP prediction of the *fabG2*, *inhA*, and *pks15 *genes and the FN prediction of the *desA3 *gene, we added the uptakes of several fatty acids from the host environment and blocked the synthesis of a fatty acid, hexadecenoate. This modification suggested that fatty acids were available in the host environment and that *M. tuberculosis *stopped synthesizing them under *in vivo *conditions. In summary, the modifications in these two groups of pathways, along with the retention of glycerol uptake (see Table 3), suggested that the survival of *M. tuberculosis *during infection in the mouse required lipids (composed of fatty acids and glycerol) instead of glucose as its primary source of carbon. The important role of lipids as carbon sources is evident from the observed up-regulation of genes involved in fatty acid catabolism during *M. tuberculosis in vivo *growth [8,28,52-55], *M. tuberculosis *growth in dipalmitoyl phosphatidylcholine (a lipid present in the mammalian lung) medium [32], the ability of the bacterium to hydrolyze lecithin into fatty acids [8,56], and the potential role of human serum as a highly effective fatty acid source [57].

In the pathways associated with "vitamin and cofactor metabolism," we removed riboflavin from the biomass objective function to correct the FP prediction of the *ribH*, *ribC*, and *ribD *genes. The removal suggested that riboflavin was not required for the survival of *M. tuberculosis *under *in vivo *conditions, which is supported by the observation that riboflavin is used for glycolysis (the pathway of glucose catabolism) in *M. tuberculosis *[58] and that glucose is not a carbon source for the pathogen in the host environment [8]. In the same pathways, we also corrected the FN predictions associated with the *bioA*, *bioF*, and *bioB *genes by adapting the biomass objective function to include biotinyl-5'-adenosine monophosphate (AMP) and blocked the ability of the gene product of the *bioF2 *gene to catalyze the synthesis of the precursor of biotin. Because biotinyl-5'-AMP is the activated form of biotin [59], the inclusion of biotinyl-5'-AMP suggests that biotin is required for the survival of *M. tuberculosis *under *in vivo *conditions. This observation is compatible with biotin playing a role in gluconeogenesis [60] and that *M. tuberculosis *obtains glucose through gluconeogenesis under *in vivo *conditions [8]. The blockade of BioF2 indicated that the enzyme may be inhibited under *in vivo *conditions, which is commensurate with the observed down-regulation of *bioF2 *in the presence of hydrogen peroxide (H_2_O_2_) [28], a reactive oxygen species that would be encountered in an intra-phagosomal environment.

In the "amino acid metabolism" pathways, we added uptakes of isoleucine and valine to correct the FP predictions of the *ilvC *and *ilvN *genes, suggesting that *M. tuberculosis *might be able to absorb these amino acids from the host environment. Although we could not directly verify these uptakes in *M. tuberculosis*, experiments have shown that a *M. tuberculosis *strain that lacks the ability to synthesize three amino acids (valine, isoleucine, and leucine) could persist in mice for four weeks [61]. This suggests that these amino acids might be available, although in limited amounts, in the host environment to compensate for the organism's inability to synthesize these amino acids.

We also examined the modifications with respect to genes and reactions involved in "transport pathways." In this group of pathways, we added two extracellular lipids, phthiocerol dimycocerosate A and phenol phthiocerol dimycocerosate, to the biomass objective function to correct the FN prediction of the *lppX *gene. The biomass objective function of *iNJ*661 and the *in vivo *biomass objective function of GSMN-TB include intracellular phthiocerol dimycocerosate A. In fact, these lipids are known to be secreted by *M. tuberculosis *into the environment and subsequently are associated with the pathogen envelope, where they aid in avoiding host immune attacks [62]. It is somewhat unusual to add extracellular metabolites to the biomass objective function; however, given the localization of the two lipids to the pathogen's envelope, they can be considered as integral to the pathogen and, hence, to the biomass. Moreover, our additions of the uptakes of NO_3_^-^, O_2_, and Fe^3+ ^(see Table 3) were supported by the existence of NO_3_^- ^in infected tissue [63], the detection of O_2 _in mouse lung granulomas [7,31], and the ability of *M. tuberculosis *to synthesize mycobactin, a chemical with a very high affinity for iron, to obtain iron from the host environment [64].

The lack of detailed experimental evidence for many modifications, as shown in Table 2, indicates that there are currently gaps of knowledge associated with *M. tuberculosis *metabolism. For example, in the transport pathways, we corrected the FN predictions of the *sugA*, *sugB*, and *sugC *genes by adding xylose uptake and including xylose in the biomass objective function. These modifications suggest that *M. tuberculosis *should have a xylose utilization pathway, although this is absent in current metabolic network descriptions of *M. tuberculosis*. The existence of such a pathway is indicated by the experimental observation that *M. tuberculosis *is able to use xylose under the presence of glycerol [65]. Conversely, although sequence analysis has suggested that the *sugABC *operon encodes a sugar-transporting system, it is unclear what sugar molecules the system transports [66], suggesting that there might be other possible reasons for the *sugABC *transport system to be essential.

### Growth prediction of double-deletion mutants

Double-gene deletion experiments provide insights into redundant pathways, non-obvious coupling of metabolite flows, and potential new drug targets. For example, although neither the *ERG11A *gene nor the *ERG11B *gene in *Aspergillus fumigatus *is individually essential, the deletion mutant of these two genes is not viable in immune-compromised mice, suggesting the pair of genes as a combined drug target [67]. Large-scale experimental double-gene deletion requires substantial efforts, whereas the corresponding *in silico *simulations of deleted gene pairs are readily available. Comparative studies for yeast (*Saccharomyces cerevisiae*) have shown that 49% of the predicted synthetically lethal double-deletion mutants are correct [68-70]. *In silico *studies of the growth of double-deletion mutants of metabolic genes have also been performed for *E. coli *[71], *Helicobacter pylori *[14], and *Leishmania major *[19], although the bulk of these predictions could not be verified due to the paucity of experimental data.

We initially examined the capability of *iNJ*661m and *iNJ*661v to model the growth of two experimentally examined double-gene deletion mutants. In the first case, experimental work showed that the growth of the *ΔpanCD *mutant (deletion of *Rv3602c *and *Rv3601c*) is highly attenuated in mice [72]. FBA of both *iNJ*661m and *iNJ*661v predicted that *ΔpanCD *mutants have a growth rate of zero, consistent with the experimental observation. Although not confirmed experimentally, our analysis of *iNJ*661m indicated that the growth attenuation may also take place within an *in vitro *medium. In the second case, experimental work has indicated that the mutant *Δicl1Δicl2 *(deletion of *Rv0467 *and *Rv1915*) of *M. tuberculosis *cannot survive in mice but can grow under certain *in vitro *conditions [32]. FBA of *iNJ*661m predicted that the growth rate of *Δicl1Δicl2 *was equal to that of wild-type *M. tuberculosis*, whereas analysis of *iNJ*661v predicted that the growth rate of *Δicl1Δicl2 *was only 26% of the wild-type rate, suggesting that *iNJ*661v was a better predictor for *in vivo *growth.

**Figure 7:**
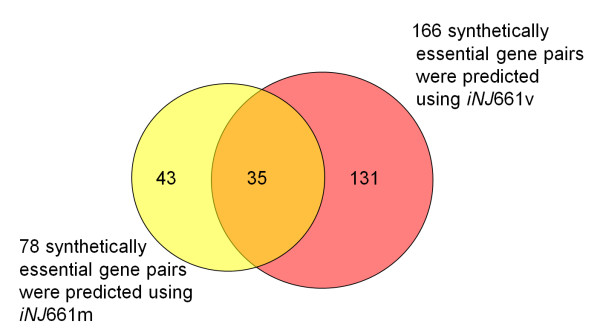
**Number of essential gene pairs predicted using *iNJ*661m and *iNJ*661v**. Flux balance analysis of *iNJ*661m under *in vivo *conditions predicted 78 essential gene pairs, whereas *iNJ*661v predicted 166 essential gene pairs. There were 35 gene pairs predicted to be essential by both network descriptions; 131 gene pairs were only predicted to be essential using *iNJ*661v, whereas 43 gene pairs were only predicted to be essential using *iNJ*661 m. Most of the jointly predicted gene pairs were involved in amino acid and nucleotide metabolism.

We then performed a comprehensive FBA of *iNJ*661m and *iNJ*661v to simulate the growth of *M. tuberculosis *double-gene deletion mutants under an *in vitro *condition and an *in vivo *condition, respectively, and predicted synthetic lethality. Additional file 1, Table S2 shows the complete results of these calculations, and Additional file 1, Figure S1 shows the mapping of synthetic essential gene pairs in *iNJ*661v to carbon metabolism-related pathways. Figure 7 shows the number of essential gene pairs that were uniquely and commonly predicted by these two networks (*iNJ*661m and *iNJ*661v). The *iNJ*661v network predicted a substantially larger number of synthetically lethal genes under *in vivo *conditions than the *iNJ*661m network under *in vitro *conditions. This was partly due to the more constrained nutritional environment faced by *iNJ*661v with respect to carbon metabolism. Although drugs effective under *in vitro *conditions may not be effective under *in vivo *conditions, the converse may also be true [9]. The additional 131 gene pairs identified in *iNJ*661v indicate novel potential drug targets under *in vivo *conditions. Likewise, the 35 gene pairs that were predicted to be essential under both conditions may represent more robust drug targets based on their insensitivity to environmental conditions.

The bulk of the 131 gene pairs uniquely predicted to be essential for bacterial growth using *iNJ*661v were related to carbon and energy metabolism. This reflects the modifications that we implemented in *iNJ*661v to reproduce the *in vivo *gene essentiality data in these parts of the metabolic network. Synthetic lethality in carbon metabolism was primarily located in the two different pathways that can be used to synthesize glucose precursors from glycerol and fatty acids (as shown by the red color in Additional file 1, Figure S1 and Table S2). Other enzyme pairs that exhibited synthetic essentiality in *iNJ*661v were those who catalyzed the same reaction, i.e., each enzyme by itself was not essential, but if they were both deleted at the same time, the reaction could not proceed and the organism would stop growing. Additional file 1, Table S2 shows that this group includes *Rv2476c*, *gltB*, and *gltD*, whose gene products were necessary for converting α-ketoglutarate into glutamate. An additional 76 synthetically essential gene pairs were involved in energy metabolism. We also analyzed the 35 synthetically essential gene pairs that were common to both *iNJ*661m and *iNJ*661v. This set was enriched in gene pairs that were involved in amino acid and nucleotide metabolism, supporting the suggestion that these metabolic processes could be common drug targets under both *in vitro *and *in vivo *conditions [34].

### Exploration of the metabolism of *M. tuberculosis *using the *in vivo iNJ*661v network

The gene essentiality data used to help construct the *in vivo *network produced non-obvious changes in the flow of metabolites and uptakes of nutrients from the environment. Analyses of this *in vivo*-compatible metabolic network of *M. tuberculosis *allow us to probe the metabolic state and metabolic adaptation of the pathogen to the host environment, opening-up new avenues for targeting specific enzymes or pathways that cannot be observed under *in vitro *conditions. Here, we briefly explored the *M. tuberculosis *metabolism as related to its adaptation to living in an acidic environment, the importance of the tricarboxylic acid (TCA)-cycle under different limiting nutrient conditions, the effects of inhibiting multiple reactions, and the modes of cellular respiration during infection.

The macrophage phagosome presents a generally hostile environment with an acidic pH ranging from 6.2 to 4.5 [73]. The specific mechanism by which *M. tuberculosis *adapts to this acidic condition has not been fully elucidated [74]. One well-known protective feature is the waxy (primarily mycolic acid) cell envelope that forms a barrier against unwanted H^+ ^entry [74]. In the context of metabolism, it is speculated that the urease reaction presents another acid adaptation mechanism by converting H^+ ^and urea into NH_4_^+ ^and CO_2 _[75,76]. The produced NH_4_^+ ^also contributes to the *M. tuberculosis *survival by preventing the maturation of the phagosome [77]. We used the *iNJ*661v network to explore additional acid adaptation mechanisms based on the metabolic flow of H^+^. We used flux variability analysis (FVA) to estimate the ranges of reaction fluxes in the metabolic network at the optimal growth rate. This allowed us to estimate the range of the overall H^+ ^exchange between the environment and the *M. tuberculosis *cells at the optimal growth rate for wild type *iNJ*661v. The protonation state of the metabolites is chosen to correspond to pH 7.2 [78]. Because we only considered the steady state H^+ ^flux in both *in vitro *and *in vivo *metabolic network models, the protonation states of the metabolites were not changed in this calculation. Table 5 shows that the overall H^+ ^exchange was within a narrow range of negative values, suggesting that the *in vivo *metabolism consumed H^+ ^as a whole, contributing to the relief of acidic stress. The primary reaction important for this H^+ ^consumption was the nitrite reductase (NR) reaction that uses H^+ ^and reduces NO_2_^- ^into NH_4_^+^. Table 5 shows that the fluxes through NR of wild type *iNJ*661v were positive but did not vary, suggesting that there must be flux through this reaction at the optimal growth of wild type cells. Conversely, for mutants whose NR was removed (denoted as ΔNR in Table 5), the overall H^+ ^exchange could only be positive. Given the neutralization as well as the protective effect of creating NH_4_^+ ^[77], NR is a strong candidate for playing an important function in the *in vivo *adaptation of *M. tuberculosis *in acidic environments.

**Table 5:** Flux ranges for overall hydrogen ion (H+) exchanges and other related reactions.

Network	Strain	**Overall H**^**+ **^**exchange** (mmol/h/gDW)		Nitrite reductase flux (mmol/h/gDW)		Urease flux (mmol/h/gDW)	
		
		Min	Max	Min	Max	Min	Max
*iNJ*661v	Wild type	-1.99	-1.93	1.00	1.00	0.00	0.00
	
	ΔNR	0.04	0.07	0.00	0.00	0.00	0.00

*iNJ*661v	Wild type	-3.96	-3.90	1.00	1.00	0.99	1.00
	
with urea uptake	ΔNR	-1.94	-1.90	0.00	0.00	0.99	1.00

*iNJ*661m	Wild type	2.36	3.09	0.00	0.00	0.00	0.00
	
	ΔNR	2.36	3.09	0.00	0.00	0.00	0.00

*iNJ*661m	Wild type	0.43	0.91	0.00	0.00	0.99	1.00
	
with urea uptake	ΔNR	0.43	0.91	0.00	0.00	0.99	1.00

Overall hydrogen ion (H^+^) exchange indicates the total H^+ ^exchange between the environment and the *Mycobacterium tuberculosis *cells. A negative value of the exchange indicates that *M. tuberculosis *consumes H^+ ^as a whole, while a positive value indicates that the metabolism generate a H^+ ^surplus, i.e., increases acidification of the environment. The minimum (Min) and maximum (Max) fluxes reflected the ranges of the fluxes at the optimal growth and were obtained through flux variability analyses of *iNJ*661v and *iNJ*661 m. ΔNR represents the strain in which nitrite reductase (NR) was blocked. The unit of mmol/h/gDW represents mmol per hour per gram dry weight of *M. tuberculosis*.

In order to more comprehensively study acid adaptation/resistance we examined the role of NR in the presence of urease [75,76]. Because the *iNJ*661 (and the GSMN-TB) network does not include a complete urea synthesis pathway, we added a urea uptake in order to create a flux through the urease reaction. Table 5 shows the estimated ranges of the H^+ ^exchange and the reaction fluxes for *iNJ*661v with urea uptake. Overall, H^+ ^is consumed to relieve acid stress. In the wild type strain, the flux through the NR reaction was greater than zero, suggesting that NR still contributes to H^+ ^consumption in the presence of urease. Removal of the NR reaction (ΔNR mutant) diminished H^+ ^consumption, but did not abolish the overall H^+ ^exchange. Importantly, we performed the same analysis for the *in vitro iN*J661m network and found that the NR reaction was always inactive, suggesting that NR does not play a role in acid adaptation in the *in vitro *medium (Table 5, last two rows). In conclusion, NR contributes to the acid adaptation under *in vivo *conditions even in the presence of other acid adaptation mechanisms. The importance of NR in acid resistance might be experimentally tested by inactivating the NR reaction and examining cellular growth under *in vivo *conditions or *in vitro *low-pH environments with freely available nitrite or nitrate.

Persistence and slow growth are clinically important states of the *M. tuberculosis *pathogen. Metabolic network models can be used to study the metabolic states associated with these conditions under a variety of conditions that mimic slow growth. In the development of the GSMN-TB model, Beste and co-workers induced slow growth by limiting the uptake of glycerol and high-lighted the corresponding changes in reaction fluxes in the glyoxylate shunt pathway [16]. Considering that fatty acids are the major carbon sources for in-host *M. tuberculosis *[32], here we simulated nutrient limitation to probe slow *in vivo *growth by reducing fatty acid uptake. We performed FVA to estimate the ranges of the fluxes through the reactions in the TCA cycle and the glyoxylate shunt pathway under slow (reduced nutrient availability) and fast (normal nutrient availability) growth. We simulated the slow *in vivo *growth by constraining fatty acid uptake of *iNJ*661v until the growth rate was one third of its original value [16], and fast growth by keeping the constraints unchanged. For each reaction we calculated the ratio of the midpoint of the flux range for slow growth to that for fast growth, where each flux was normalized to the corresponding total growth rate. This ratio represents a relative value of the fluxes and can be used to compare the relative importance of specific reactions.

**Figure 8:**
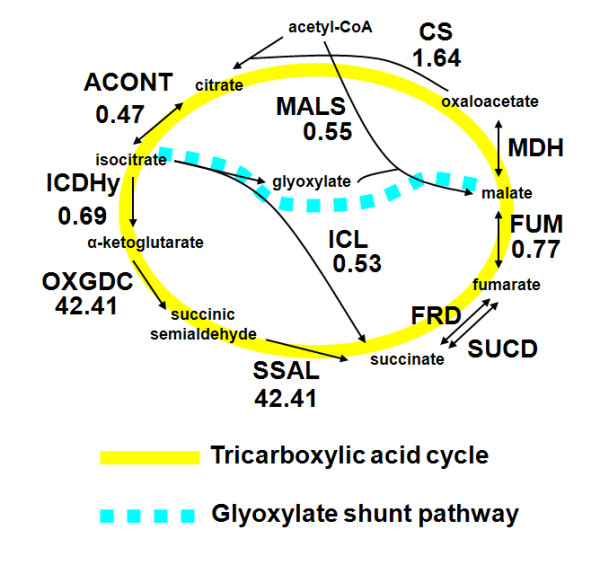
**Metabolic responses of the *iNJ*661v network to fatty-acid-limited growth**. Metabolite flow was characterized for enzymes in the tricarboxylic acid cycle and the glyoxylate shunt pathway. The numbers in the graph indicate ratios of normalized flux-range midpoints. These were calculated based on flux variability analysis for slow and fast growth conditions, where the fluxes were normalized by dividing by the corresponding total growth rates. This normalization removes artifacts introduced by the lower absolute reaction fluxes associated with induced slow growth [16]. CS, citrate synthase; ACONT, aconitase; ICDHy, isocitrate dehydrogenase; OXGDC, 2-oxoglutarate decarboxylase; SSAL, succinate-semialdehyde dehydrogenase; FRD, fumarate reductase; SUCD, succinate dehydrogenase; FUM, fumarase; MDH, malate dehydrogenase; MALS, malate synthase.

Figure 8 shows the relevant metabolites and enzymes and highlights the significantly decreased ratios in the glyoxylate shunt pathway and increased reaction fluxes in parts of the TCA cycle. The increased reaction fluxes in *iNJ*661v included the 2-oxoglutarate decarboxylase (OXGDC) and succinate-semialdehyde dehydrogenase (SSAL) reactions, suggesting that these reactions were likely to be important in fatty-acid-limited slow growth. The previously suggested increased flux and importance of isocitrate lyase (ICL) under slow-growth conditions is a direct consequence of the glycerol limitation [16]. In fatty-acid-limited slow growth, which is consistent with the *in vivo *metabolic state, the OXGDC and SSAL reactions took on a heightened function and importance in slow-growth maintenance. The suggested metabolic responses to fatty-acid-limited growth rate could be experimentally tested by measuring the activities of the enzymes in the TCA cycle of *M. tuberculosis *during slow and normal growth in host environment or in an *in vitro *condition infused with fatty acids as carbon sources.

**Figure 9:**
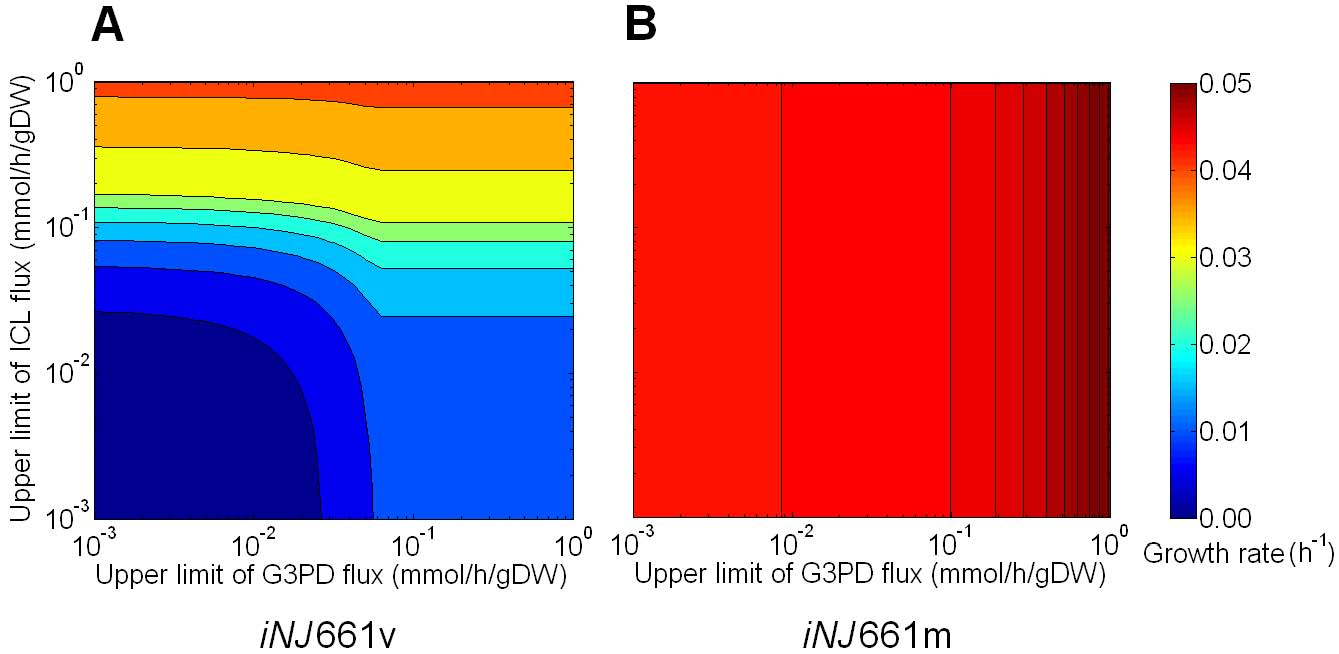
**Predicted effects of a double-reaction inhibition on the *in vivo *growth of *Mycobacterium tuberculosis***. The two inhibited reactions were isocitrate lyase (ICL) and glycerol-3-phosphate dehydrogenase (G3PD). The growth rates (in units of h^-1^) were calculated based on flux balance analysis with different upper limits of the fluxes through the two reactions. The upper limits are in unit of mmol/h/gDW, i.e., mmol per hour per gram dry weight of *M. tuberculosis*. *Panel A *shows the results of the combinational inhibition using the *iNJ*661v network; *panel B *shows the *in vitro iNJ*661m results.

Given the ability of the metabolic network to provide different reaction fluxes under different metabolic conditions, we can explore combinations of mechanisms to inhibit multiple reactions to derive optimal *in vivo *growth-reduction strategies. Drug combinations that achieve optimal therapeutic response and avoid side effects caused by high doses of single drugs [79,80] can rapidly be examined using these modeling techniques. To illustrate this concept, we constructed an example to investigate double-reaction inhibition using metabolic network modeling. Given that the primary *in vivo *nutrients are lipids, we focused on two reactions that are required to process these metabolites, i.e., the glycerol-3-phosphate dehydrogenase (G3PD) reaction, which is necessary for the utilization of glycerol, and the ICL reaction, which is a known potential drug target and is required for the conversion of fatty acids into other metabolites, such as pyruvate [32]. To study the effect of this combined inhibition, we calculated growth rates under a set of upper limits of the fluxes through the ICL and G3PD reactions. Figure 9 shows the calculated growth rates under different upper limits for the two reactions for both the *in vivo iNJ*661v (*panel A*) and *in vitro iNJ*661m (*panel B*) networks.

In these graphs, the lower left hand corner corresponds to the most stringent blockage of both reactions, i.e., where the fluxes for each reaction was constrained to be ≤10^-3 ^mmol/h/gDW. Figure 9A, illustrating the *in vivo *results, shows that these flux limits effectively blocked growth of the organism as indicated by the blue color designating strongly retarded growth rates. One can also see that whereas the ICL reaction is essential, i.e., the organism cannot effectively grow if this reaction is sufficiently inhibited, single blockage of the G3PD reaction would not prevent growth if the ICL reaction was left unconstrained (top portion of Figure 9A). It was also clear that limiting the G3PD flux (i.e., going from right to left in Figure 9A) further decreased the growth rate at virtually all ICL flux values. This indicates that for a given desired growth-rate reduction, one could use a combination of ICL and G3PD inhibitions to achieve the same effect as those possible with a stringent single ICL inhibition. If these fluxes can be inhibited by drugs, reducing the dose of the most toxic drug to achieve the same therapeutic response would decrease risk of potential side effects. Moreover, we performed the same calculation for *iNJ*661m and observed no combinatorial effect of the inhibition. Figure 9B shows that when we constrained the fluxes through the ICL and G3PD reactions to zero, the growth rate was still close to that of unconstraint growth. It is clear that the *iNJ*661v network is instrumental in delineating combinatory inhibition strategies while the *in vitro iNJ*661m network is not.

Finally, we examined the results of the *iNJ*661v double deletion mutants with respect to cellular respiration during infection. Additional file 1, Table S2 shows that the genes corresponding to the cytochrome bd oxidase (*cydB-cydD *and *appC*) and the nitrate reductase (*narG-narJ*) enzymes were synthetically essential. Because these two enzymatic groups are associated with aerobic and anaerobic respiration, respectively, synthetic essentiality indicates that therapy targeting cellular respiration needs to simultaneously inhibit both aerobic and anaerobic respiration. This conclusion is not evident from gene expression data, which instead indicates that seven weeks post-infection the pathogen only employs nitrate reductase enzymes for anaerobic respiration [81]. However, gene essentiality data indicate that all genes corresponding to these enzymes (*narG-narJ*) are non-essential in mice, even after seven weeks [34], indicating that anaerobic respiration is not the only available option to the pathogen and that O_2 _is available to the bacterium in the host environment [31]. Therefore, dual inhibition of aerobic and anaerobic is necessary to fully arrest bacterial respiration. This hypothesis can be experimentally tested by simultaneously deleting both gene groups and testing for differential growth of *M. tuberculosis *under *in vivo *and *in vivo *conditions.

In summary, we used the developed *iNJ*661v network to explore metabolism-based *M. tuberculosis *acid adaptation mechanisms, study metabolic responses to *in vivo *slow growth, estimate the effects of different levels of inhibitions of multiple reactions, and gain insights into respiration-targeting therapy.

## Discussion

Although the existing metabolic network of *M. tuberculosis iNJ*661 [15] reproduces experimentally observed growth rates in different media and predicts gene essentiality under *in vitro *conditions, it lacks predictive power for *in vivo *gene essentiality. We developed a set of automated procedures that systematically examined possible metabolic modifications to the original *iNJ*661 network and optimized predictions of experimental *in vivo *essentiality. By design, the newly developed *in vivo *network *iNJ*661v provided significantly better agreement (sensitivity increased from 0.31-0.44 to 0.81-0.86; Table 1) between predicted and experimental *in vivo *gene essentiality.

Indirectly, the systematic reconstruction of *iNJ*661v also provided a means to use high-throughput *in vivo *gene essentiality data to gain insights into the *in vivo *metabolism of *M. tuberculosis*. For example, we added fatty acids as inputs from the host environment and removed the ability of the network to take up glucose, indicating that *M. tuberculosis *uses lipids as the major carbon source for metabolism. This is compatible with the experimentally observed upregulation of genes involved in fatty acid catabolism [8,28,52-55]. Commensurate with the aerobic nature of the organism, our calculations indicated that O_2 _uptake must be retained under *in vivo *conditions [7,31,82]. Another example is our inclusion of two extracellular lipids, phthiocerol dimycocerosate A and phenol phthiocerol dimycocerosate, in the biomass objective function. This is appropriate since the two secreted lipids actually associate with the pathogen envelope to help the organism avoid host immune attacks [62] and are thus intimately associated with the organism/cell itself. In addition to providing experimentally confirmed insights, the *iNJ*661v network allowed us to predict a different and putative much larger set of synthetic double-gene deletion mutants than those obtained under *in vitro *conditions. Furthermore, using *iNJ*661v as a model for *in vivo *metabolism, we proposed that nitrite reductase might play an important role in the metabolic adaptation of *M. tuberculosis *to an acidic environment, we found specific enzymes in the TCA-cycle that might be important under the fatty-acid-limiting slow-growth condition prevailing in macrophages, we investigated combinatory inhibition of the ICL and G3PD reactions as an effective drug-combination strategy under *in vivo *nutrient conditions, and found that inhibition of both aerobic and anaerobic respiration were required to fully arrest cellular respiration of *M. tuberculosis *during infection.

The developed methodology can provide systematic corrections based primarily on discrepancies between predicted and experimental gene essentiality data, which can be used to fine-tune initial metabolic network reconstructions. Here, we built on and expanded the previously developed "GrowMatch" techniques of Kumar and Maranas [39] by extending and enhancing the possible correction steps. This was partly necessitated by the more incomplete state of the *M. tuberculosis *network compared with that of *E. coli*, as used by Kumar and Maranas, and the more extensive nature of the required corrections to switch from an *in vitro *to an *in vivo *metabolic environment. One major difference was that our procedures included an analysis of the combined modifications (*Step III *in Figure 1, with details shown in Figure 5) to systematically eliminate undesirable effects, e.g., very small growth rates or new incorrect predictions of gene essentiality.

Although the gene deletion mutant growth measurements used by Sassetti and Rubin [34] to experimentally determine gene essentiality provide time-specific information, the developed *iNJ*661v network did not include a time-dependent component. In the development of our network, a gene was considered experimentally essential as long as it was deemed to be essential at any time point during the entire eight-week time course spanned by the experiments [34]. We examined this approximation by comparing gene essentiality predictions at the reported time points of one, two, four, and eight weeks post-infection. Additional file 1, Table S3 shows a comparison of the predicted and experimental gene essentiality at different time points, basically showing that there was an overall small, non-time-specific difference between the MCC values for the individual time points and the time-independent value (0.47) shown in Table 1. The sparse nature of the experimental data did not warrant the additional complexity of constructing time-dependent *in vivo *metabolic networks. However, as both the pathogen and host dynamically change their responses during infection, future work using additional datasets and modeling methodologies will be required to adequately capture this aspect of the *in vivo *metabolism of *M. tuberculosis*.

The existence of inaccurate essentiality predictions based on *iNJ*661v (FN and FP; Table 1) indicate that there is room for further additions and corrections to the network to better capture *in vivo *metabolism. The modifications that we made to the original *in vitro iNJ*661 network were relatively minor, as *iNJ*661v only shows a slightly diminished capability to predict *in vitro *essentiality data compared with *iNJ*661 m. The development of the modified *in vivo *network *iNJ*661v was ultimately based on the growth of the bacterium under different *in vitro *conditions (*iNJ*661) and modifications to better model experimental *in vivo *gene essentiality. These conditions capture part of the pathogen's metabolic processes, but large knowledge gaps still exist. An analysis of the *M. tuberculosis *genome indicated that while 1,286 genes are directly associated with metabolic processes (C. Yu, personal communication), only 663 genes were explicitly included in the developed network. This highlights the need for further systematic theoretical analyses to improve the network description and, more importantly, the need for experimental data under a variety of different *in vivo *and *in vitro *growth conditions that could be used to guide and validate model development.

Higher fidelity *in silico *modeling of organisms provides the foundation for the eventual integration of metabolic information with gene regulation and signaling networks to model biological phenomena. Ultimately, the model development presented here can be extended to answer additional questions as they relate to the metabolic status of the pathogen population before, during, and after infection: What nutrients are present in different *in vivo *compartments, how do they change as infection progresses, and how do they relate to cellular growth rates and population sizes? What is the appropriate objective function to use for the persistent dormant phase of *M. tuberculosis *infection? What processes does *M. tuberculosis *use to handle nutrient deficiencies and antagonistic conditions found in macrophages? These are the questions that we can now begin to address using the developed *iNJ*661v as a more sophisticated *in vivo *representation of the metabolic network for *M. tuberculosis*.

## Conclusion

*M. tuberculosis*, the causative agent of TB, continues to pose a major health threat worldwide, with nearly two million deaths annually. Modeling of and accounting for the varying metabolic requirements of *M. tuberculosis *during host infection can help identify the metabolic enzymes suitable for therapeutic intervention. To this end, we developed procedures to construct an *in vivo *metabolic network model of *M. tuberculosis *that maximizes the agreement between predicted and measured gene essentiality determined from infection experiments in the mouse. We verified the modifications obtained computationally by reviewing the available relevant literature. For example, lipids are major carbon sources for *M. tuberculosis *in the host environment. The network provided a metabolic description of the pathogen consistent with the generally hostile and nutrient-poor *in vivo *conditions in the host that can be exploited in evaluation, selection, and modeling of novel potential drug targets.

## Authors' contributions

All authors contributed to the design and coordination of the study. XF performed the computational implementations, and XF and AW prepared the original draft, which was revised by JR. All authors read and approved the final manuscript.

## Supplementary Material

Additional file 1 **Supplementary materials**. It provides the detailed steps for constructing the *iNJ*661m and *iNJ*661v networks, and all supplemental tables and figure. Click here for file

Additional file 2 **The *iNJ*661m metabolic network**. It describes the *iNJ*661m metabolic network, using the Systems Biology Markup Language format. Click here for file

Additional file 3 **The *iNJ*661m metabolic network**. It describes the *iNJ*661m metabolic network, using Microsoft Excel 2003. Click here for file

Additional file 4 **The *iNJ*661v metabolic network**. It describes the *iNJ*661v metabolic network, using the Systems Biology Markup Language format. Click here for file

Additional file 5 **The *iNJ*661v metabolic network**. It describes the *iNJ*661v metabolic network, using Microsoft Excel 2003. Click here for file
