# Supplementary material for: Development and analysis of an in vivo-compatible metabolic network of Mycobacterium tuberculosis
Source: BMC Syst Biol. 2010 Nov 23;4:160. doi: 10.1186/1752-0509-4-160 (PMC3225870; doi:10.1186/1752-0509-4-160)
Supplement: Additional file 1 — Supplementary materials. It provides the detailed steps for constructing the iNJ661m and iNJ661v networks, and all supplemental tables and figure. [file 1752-0509-4-160-S1.PDF]

Supplemental Material

Development and analysis of an *in vivo*-compatible metabolic network of *Mycobacterium tuberculosis*

Xin Fang, Anders Wallqvist, Jaques Reifman[§]

Biotechnology HPC Software Applications Institute, Telemedicine and Advanced Technology Research Center, U.S. Army Medical Research and Materiel Command, Ft. Detrick, MD 21702, USA

[§]Corresponding author

Email addresses:

XF: xfang@bioanalysis.org

AW: awallqvist@bioanalysis.org

JR: jaques.reifman@us.army.mil

Index

S1. Development of the *iNJ*661m network ……………. 3

S2. Detailed description of the computational procedures used to develop the *iNJ*661v network………………..... 8

S3. Supplemental Tables and Figure ………………..... 20

Supplemental Table S1 ……………………………………………….…...... 20

Supplemental Table S2 ……………………………………………….…...... 24

Supplemental Table S3 ……………………………………………………… 35

Supplemental Figure S1 …………………………………………………….. 36

S1. Development of the *iNJ*661m network

Biotin is an important cofactor for metabolism; however, biotin synthesis in *iNJ*661 is not connected to other pathways in the network. To remedy this, we inserted two related reactions from GSMN-TB [1], added four small molecules (H_2O, H^+, NH_4^+, and O_2) not accounted for by GSMN-TB into the two reactions, and inserted those reactions into the *iNJ*661m network. The first reaction provides for the synthesis of a precursor of biotin, pimeloyl-CoA, from acetyl-CoA:

H_2O + 4 acetyl-CoA → pimeloyl-CoA + 3 CoA + CO_2 + H^+. (1)

In the biotin synthesis pathway, the reaction of adenosylmethionine-8-amino-7-oxononanoate transaminase converts *S*-adenosyl-L-methionine to *S*-adenosyl-4-methylthio-2-oxobutanoate. However, *iNJ*661 lacks a necessary reaction that does the opposite, and we needed to add the following reaction to *iNJ*661m:

2 NH_4^+ + 2 *S*-adenosyl-4-methylthio-2-oxobutanoate → 2 *S*-adenosyl-L-methionine + O_2. (2)

According to the naming convention of enzymes, succinate dehydrogenase dehydrogenates succinate into fumarate, and fumarate reductase reduces fumarate into succinate. The functions of these two enzymes in the metabolic network of *Escherichia coli* follow this enzyme naming convention [2]. However, the reactions catalyzed by these two enzymes in *iNJ*661 have discrepancies in their directions. In *iNJ*661, succinate dehydrogenase catalyzes the following reactions:

fumarate + menaquinol 8 → menaquinone 8 + succinate, (3)

fumarate + 2-demethylmenaquinol 8 → 2-demethylmenaquinone 8 + succinate, and (4)

succinate + FAD → $FADH_2$ + fumarate, (5)

where the first two reactions have the opposite directions with respect to fumarate as the last reaction. Fumarate reductase catalyzes the following reactions:

fumarate + $FADH_2$ → FAD + succinate, and (6)

fumarate + menaquinol 6 ↔ menaquinone 6 + succinate, (7)

one of which is irreversible and one which is reversible. In the KEGG database, the conversion between succinate and fumarate in *Mycobacterium tuberculosis* is a reversible reaction that is catalyzed by either succinate dehydrogenase or fumarate reductase (EC-1.3.99.1) [3]. In GSMN-TB, both enzymes catalyze reversible conversions between succinate and fumarate [1]. Furthermore, the metabolic networks of *Pseudomonas aeruginosa* [4], *Staphylococcus aureus* [5], and *Saccharomyces cerevisiae* [6] include only succinate dehydrogenase catalyzing a reversible conversion between succinate and fumarate. Consequently, we altered the reactions of *Eqs. 3-7* to be reversible in the *iNJ*661m network.

The methylcitrate cycle exists in *M. tuberculosis* [7, 8], but it is not included in *iNJ*661. We added this pathway to *iNJ*661m by inserting two new metabolites, methylcitrate and methylisocitrate, and the following reactions:

propionyl-CoA + H_2O + oxaloacetate → methylcitrate + CoA + H^+, (8)

methylcitrate ↔ methylisocitrate, and (9)

methylisocitrate → succinate + pyruvate. (10)

The first reaction is catalyzed by the gene product of *gltA1* (*Rv1131*) [8], the second reaction is catalyzed by the gene products of both *Rv1130* and *acn* (*Rv1475c*) together [8], and the third reaction is catalyzed by either the product of gene *icl1* (*Rv0467*) or that of gene *icl2* (*Rv1915*) [7-9]. The *iNJ*661 network already includes enzymes from four (*Rv1131, acn,*

icl1, and *icl2*) of the five genes above in reactions other than *Eqs. 8-10*. Because *Rv1130* is not included, we added this gene to the *iNJ*661m network. Moreover, it has been reported that when vitamin B_{12} is present in the medium, the methylmalonyl pathway can bypass the methylcitrate cycle [10]. Because the *in vitro* medium studied here did not include this vitamin [11], we blocked the methylmalonyl pathway in *iNJ*661m.

From the TubercuList World-Wide Web Server [12], we found that the gene products of both *fadB3* (*Rv1715*) and *fadB2* (*Rv0468*) yield the same β-hydroxybutyryl-CoA dehydrogenase enzyme. However, *fadB3* is not included in *iNJ*661, and, consequently, we added *fadB3* to *iNJ*661m to serve as an alternative for *fadB2*.

M. tuberculosis grows in several different fatty acid media [9], suggesting that the pathogen is able to absorb different fatty acids from the host environment. However, uptake reactions for only three fatty acids (propionate, hexadecanoate, and octadecanoate) exist in *iNJ*661, so we added uptake reactions for other fatty acids to *iNJ*661m.

During the construction of *iNJ*661m, we slightly modified the biomass objective function from the original *in vitro* network [11]. Ions and cofactors required for cellular growth can be included in a biomass objective function [2, 13], and, because both Na^+ and K^+ play important roles in *M. tuberculosis* biochemical activities [14, 15], we included these ions in the biomass objective function. We set the coefficients for the two metabolites to 10^{-6} mmol/gram dry weight, that is, 10^{-6} millimoles per gram dry weight of *M. tuberculosis*. This is the same value as the one used in the *iNJ*661 network for vitamins and cofactors [11].

References

1. **Beste DJ, Hooper T, Stewart G, Bonde B, Avignone-Rossa C, Bushell ME, Wheeler P, Klamt S, Kierzek AM, McFadden J: GSMN-TB: a web-based genome-scale network model of Mycobacterium tuberculosis metabolism.** ***Genome Biol*** **2007, 8:R89.**

2. **Feist AM, Henry CS, Reed JL, Krummenacker M, Joyce AR, Karp PD, Broadbelt LJ, Hatzimanikatis V, Palsson BO: A genome-scale metabolic reconstruction for Escherichia coli K-12 MG1655 that accounts for 1260 ORFs and thermodynamic information.** ***Mol Syst Biol*** **2007, 3:121.**

3. **Kanehisa M, Araki M, Goto S, Hattori M, Hirakawa M, Itoh M, Katayama T, Kawashima S, Okuda S, Tokimatsu T, Yamanishi Y: KEGG for linking genomes to life and the environment.** ***Nucleic Acids Res*** **2008, 36:D480-484.**

4. **Oberhardt MA, Puchalka J, Fryer KE, Martins dos Santos VA, Papin JA: Genome-scale metabolic network analysis of the opportunistic pathogen Pseudomonas aeruginosa PAO1.** ***J Bacteriol*** **2008, 190:2790-2803.**

5. **Becker SA, Palsson BO: Genome-scale reconstruction of the metabolic network in Staphylococcus aureus N315: an initial draft to the two-dimensional annotation.** ***BMC Microbiol*** **2005, 5:8.**

6. **Duarte NC, Herrgard MJ, Palsson BO: Reconstruction and validation of Saccharomyces cerevisiae iND750, a fully compartmentalized genome-scale metabolic model.** ***Genome Res*** **2004, 14:1298-1309.**

7. **Gould TA, van de Langemheen H, Munoz-Elias EJ, McKinney JD, Sacchettini JC: Dual role of isocitrate lyase 1 in the glyoxylate and methylcitrate cycles in Mycobacterium tuberculosis.** ***Mol Microbiol*** **2006, 61:940-947.**

8. **Munoz-Elias EJ, Upton AM, Cherian J, McKinney JD: Role of the methylcitrate cycle in Mycobacterium tuberculosis metabolism, intracellular growth, and virulence.** ***Mol Microbiol*** **2006, 60:1109-1122.**

9. **Munoz-Elias EJ, McKinney JD: Mycobacterium tuberculosis isocitrate lyases 1 and 2 are jointly required for in vivo growth and virulence.** ***Nat Med*** **2005, 11:638-644.**

10. **Savvi S, Warner DF, Kana BD, McKinney JD, Mizrahi V, Dawes SS: Functional characterization of a vitamin B12-dependent methylmalonyl pathway in Mycobacterium tuberculosis: implications for propionate metabolism during growth on fatty acids.** ***J Bacteriol*** **2008, 190:3886-3895.**

11. **Jamshidi N, Palsson BO: Investigating the metabolic capabilities of Mycobacterium tuberculosis H37Rv using the in silico strain iNJ661 and proposing alternative drug targets.** ***BMC Syst Biol*** **2007, 1:26.**

12. Tuberculist Web Server, Pasteur Institute. http://genolist.pasteur.fr/TubercuList/

13. Feist AM, Herrgard MJ, Thiele I, Reed JL, Palsson BO: Reconstruction of biochemical networks in microorganisms. *Nat Rev Microbiol* 2009, 7:129-143.

14. Hase CC, Fedorova ND, Galperin MY, Dibrov PA: Sodium ion cycle in bacterial pathogens: evidence from cross-genome comparisons. *Microbiol Mol Biol Rev* 2001, 65:353-370, table of contents.

15. Epstein W: The roles and regulation of potassium in bacteria. *Prog Nucleic Acid Res Mol Biol* 2003, 75:293-320.

S2. Detailed description of the computational procedures used to develop the iNJ661v network

The procedures and analyses described below (*A*-*F*) capture the logical flow of creating and testing the metabolic network modifications, while the optimization schemes (*G* and *H*) detail the specific modifications we made to the optimization models of Kamal and Maranas (Ref. 39 in the main text).

A. **Main procedure** (Figure 1 in the main text)
B. **FP correction procedure** (Figure 2 in the main text)
C. **FN correction procedure** (Figure 3 in the main text)
D. **Assessment of a modification** (Figure 4 in the main text)
E. **Analysis of combined modifications** (Figure 5 in the main text)
F. **Nutrient uptake analysis**
G. **Optimization scheme 1: FP corrections**
H. **Optimization scheme 2: FN corrections**

A. **Main procedure** (Figure 1 in the main text)

Identify incorrect predictions of gene essentiality
For each incorrect prediction
    If   (the prediction is a false positive (FP) prediction)
        FP correction procedure
    Else
        FN correction procedure
    End_if
End_for
Analysis of combined modifications
Nutrient uptake analysis
Literature verification

B. FP correction procedure (Figure 2 in the main text)

//Examine consequences of deleting metabolites from the biomass [Figure 2 (*I*)]
NM = 1
// NM represents the number of metabolites deleted from biomass
NF = 0
// NF=0, no modification has been found, NF = 1 modification exists
While (NF = 0 AND NM $\leq$ 16)
    List all possible deletions of NM metabolite(s) from the 16 vitamins and cofactors
    //This causes all single deletions to be considered first, then all possible double
    //deletions, etc.
    For each deletion
        If (the deletion is adequate - see **Assessment of a modification**)
            Record the deletion
            NF = 1
            //If a satisfactory modification is recorded, we will end the
            //loop after considering all NM deletions
        End_if
    End_for
    NM = NM + 1
End_while
//Make irreversible reactions reversible and allow additional nutrient uptakes [Figure 2 (*II*)]
//
//The goal here is to find modification(s) compatible with changing a minimum number of
//reversible reactions and allowing a minimum number of nutrient uptakes to correct the FP
//predictions. To implement **Optimization scheme 1: FP corrections**, we need to
//specify *1*) a set of reactions P that are potentially blocked or added to the network, *2*)
//exactly what the function is that is minimized, and *3*) what the growth cutoff is:

Let *P* contain both blocked uptake reactions (*P1*) and irreversible reactions with their directions reversed (*P2*)
Set the function to be minimized to be $\min \sum_{j\in P1} y_j + 100 \sum_{j\in P2} y_j$
//where *j* is a reaction index and *y* is binary variable (0/1) indicating whether the reaction is
//included or no. Since it is less desirable to change the direction of a reaction, we penalized
//these modifications by a factor of 100.
Set the cutoff $v_{biomass}^{cut-off}$ to $0.2\, v_{biomass}^{wild-type}$
//where $v_{biomass}^{wild-type}$ represents the wild-type growth rate and was obtained flux balance
//analysis (FBA) of iNJ661m.

Optimization scheme 1: FP corrections

//The minimization model may generate more than one solution, and we need to consider
//each solution
For each modification found from the optimization model
    If (the modification is adequate - see **Assessment of a modification**)
        Record the modification
    End_if
End_for

C. FN correction procedure (Figure 3 in the main text)

Find all reactions catalyzed by the product of the FN gene
For each reaction
// Check whether the reaction is associated with another TN gene(s)
If [the FN gene and TN gene(s) are jointly necessary for the reaction]
The prediction can not be corrected for: Go to the next reaction
End_if
// Check whether FN gene product has one or more isozyme(s)
If [isozyme(s) of the FN gene product catalyze the reaction]
Block the ability of isozyme(s) to catalyze the reaction
If (the blocking is adequate - see **Assessment of a modification**)
Record the modification
End_if
End_if
// Check whether the FN gene is in a dead-end pathway
If [the reaction is in a pathway where some metabolite(s) cannot be produced or consumed]
If (metabolite cannot be produced)
If [there exists blocked uptake reaction for the metabolite(s)]
Record allowing the uptake of the metabolite(s)
End_if
End_if
If (metabolite cannot be consumed)
Record adding the metabolite(s) to biomass
End_if

If (the modifications are adequate - see **Assessment of a modification**)
//The modification includes all cumulative changes to the studied reaction,
//including the isozyme(s) blocking
Record the modification and go to the next reaction
//If adequate medications are found the next section of suppressing reactions
//is not performed
End_if
End_if
//Suppress reaction(s)
//The goal here is to find modification(s) associated with suppressing a minimum
//number of reactions using **Optimization scheme 2: FN corrections**.

//To implement **Optimization scheme 2: FN corrections**, we need to
//specify *1*) a set of reactions P that are potentially blocked and *2*) what the growth
//cutoff $v_{biomass}^{cut-off}$ is:

Let P contain all reactions in *iNJ*661m
Set the growth rate limit for gene knockout to be $0.2\, v_{biomass}^{wild-type}$
//where $v_{biomass}^{wild-type}$ represents the wild-type growth rate as obtained from FBA of
//iNJ661m. A FN prediction becomes a TN prediction if the growth rate falls below
//this limit.

Optimization scheme 2: FN corrections

```
//The optimization procedure may generate more than one solution, and we need to
//consider each
For     (each modification found from the optimization model)
        If      (the modification is adequate - see Assessment of a modification)
                Record the modification and go to the next reaction
        End_if
End_for
End_for
```

D. **Assessment of a modification** (Figure 4 in the main text)

```
Apply the modification to iNJ661m
Calculate wild-type growth rate from FBA of the modified network
If      (wild-type growth rate ≤ minimal growth rate)
// Note: minimal rate = 0.027 h^-1
        The modification is inadequate
Else
        Delete the gene for the prediction from iNJ661m with the modification
        Calculate growth rate of the gene knockout
        //This case checks modifications generated by the FN correction procedure
        If      (the prediction is FN)
                If      (knockout growth rate > wild-type growth rate *20%)
                        The modification is inadequate
                Else
                        If  (any TN prediction becomes FP or any TP prediction becomes FN)
                                The modification is inadequate
                        Else
                                The modification is adequate
                        End_if
                End_if
        End_if
        //This case checks modifications generated by the FP correction procedure
        If      (the prediction is FP)
                If      (knockout growth rate ≤ wild-type growth rate *20%)
                        The modification is inadequate
                Else
                        If  (any TN prediction becomes FP or any TP prediction becomes FN)
                                The modification is inadequate
                        Else
                                The modification is adequate
                        End_if
                End_if
        End_if
End_if
```

E. **Analysis of combined modifications** (Figure 5 in the main text)

```
List all the network realizations of the modifications:
//Here, each network realization contains one modification for each incorrect prediction
For     each network realization
        If      (any contradiction between modifications in the realization)
                The network realization is inadequate
        Else
                Apply the modifications in to iNJ661m
                If      (wild-type growth rate ≤ minimal rate)
                //Note: minimal rate = 0.027 h^-1
                        The network is inadequate
                Else
                        If      (incorrect predictions become correct)
                                If      (any TP prediction becomes FN or any TN prediction
                                        becomes FP)
                                        The network is inadequate
                                Else
                                        Record the network as plausible
                                End_if
                        Else
                                The network is inadequate
                        End_if
                End_if
        End_if
End_for
Select networks with minimum adjustment from the set of plausible networks
//A minimum adjustment indicates a minimum number of irreversible reactions changed and
//reactions suppressed
Record selected networks as adequate network realizations
```

F. Nutrient uptake analysis

We only allowed selected nutrient uptakes that defined a minimal uptake set:

(1) Uptakes of H_2O and H^+

(2) Uptakes added in the **FP correction procedure** and **FN correction procedure**

(3) Uptakes of small molecules:

CO and CO_2 for carbon (C);
NH_4^+, NO_2^-, and NO_3^- for nitrogen (N);
O_2 for oxygen (O);
HPO_4^{2-} for phosphorus (P);
SO_4^{2-} for sulphur (S);
Fe^{2+} and Fe^{3+} ions for iron (Fe);
K^+ for potassium (K);
Na^+ for sodium (Na).

We defined the extended set as uptake reactions that are not in the minimal set
//The small-molecule set was selected based on existing uptake reactions in iNJ661m and
//chosen such that each molecule contained one specific element of the elements found in
//the biomass objective function and the minimum number of other elements.

//Here, we sequentially corrected the proposed minimal uptake set if it:
// 1) failed to maintain growth
// 2) generated new FP predictions
// 3) generated new FN predictions

// 1) Failure to maintain growth
If (wild-type growth rate < minimal rate)
//Note: minimal rate = 0.027 h^{-1}
//The goal here is to add a minimum number of uptakes from the extended set using
//**Optimization scheme 1: FP corrections**

Let *P* contain the extended reaction set defined above
Set the cutoff $v_{biomass}^{cut-off}$ to the minimal rate (0.027 h^{-1})

//However, as no specific FP gene is considered, the constraint given in *Equation 2*
//of the optimization scheme is removed
Optimization scheme 1: FP corrections
End_if

//2) Generation of new FP predictions
If (new FP prediction is generated)
//The goal here is to add a minimum number of uptakes from the extended set using
//**Optimization scheme 1: FP corrections**

Let *P* contain the extended reaction set defined above
Set the cutoff $v_{biomass}^{cut-off}$ to $0.2\, v_{biomass}^{wild-type}$
//where $v_{biomass}^{wild-type}$ represents the wild-type growth rate and was obtained by FBA of
//iNJ661m
Optimization scheme 1: FP corrections
End_if

//3) Generatation of new FN predictions
If (new FN prediction is generated)
//The goal here is to find modification(s) associated with suppressing a minimum
//number of reactions using **Optimization scheme 2: FN corrections**.

//To implement **Optimization scheme 2: FN corrections**, we need to
//specify *1*) a set of reactions P that are potentially blocked and *2*) what the growth
//cutoff $v_{biomass}^{cut-off}$ is.

Let *P* contain all reactions in the minimal set defined above
Set the growth rate limit for gene knockout to be $0.2\, v_{biomass}^{wild-type}$
//where $v_{biomass}^{wild-type}$ represents the wild-type growth rate as obtained from FBA of
//iNJ661m. A FN prediction becomes a TN prediction if the growth rate falls below
//this limit.
Optimization scheme 2: FN corrections
End_if
//
// Find the minimal set of small-molecule uptakes
//
//Analyze each set of small-molecule uptakes, based on the C, N, O, S, P, K, and Na
//sets defined above, to find the minimum number of uptakes or combinations of
//uptakes from each set that can maintain wild-type growth and not generate any additional
//FP or FN predictions
For each element of C, N, O, S, P, K, and Na
NSU = 0
//NSU represents the number of small molecules whose uptakes are
//allowed
UR = 0
//UR=0: no small molecule selection is recorded
//UR=1: a small molecule selection(s) has been recorded
While (UR = 0)
List all possible selection(s) of NSU small molecule(s) for the element
//For example, if the element is N and NSU = 1, the possible selections are
//NH_4^+, NO_2^-, and NO_3^-; if the element is N and NSU = 2, the possible
//selections are NH_4^+ and NO_2^-, NH_4^+ and NO_3^-, and NO_2^- and NO_3^-
For each possible selection
Only allow uptakes of small molecules containing the element in the selection (other small molecule uptakes for this particular element are blocked) while other small molecule uptakes for other elements are allowed
If (wild-type growth rate > minimal rate) AND
(no new FP or FN prediction)
Record the selection
UR = 1
End_if
End_for
NSU = NSU + 1
End_while

End_for
List all possible combinations of the small-molecule selection(s)
//For each element, list the each possible selections and create a list of all possible
//combinations of these selections. For example, if there is one selection possible for
//element *X* and two selections possible for element *Y*, there are in total two ways these can
//be combined
For each combined small-molecule selection
    Among small-molecule uptakes, only allow uptakes of the small molecules in the selection
    If (wild-type growth rate > minimal rate) AND (no new FP or FN prediction)
        Record the combined small-molecule selection as adequate
    End_if
End_for
If no combined small-molecule selection is recorded as adequate, we restore all small-molecule uptakes

G. Optimization scheme 1: FP corrections

This optimization scheme attempts to restore the biomass accumulation rate (or growth rate) $v_{biomass}$ to be above a given cutoff $v_{biomass}^{cut-off}$ by adding a minimum number of reactions to a network. If *P* represent the candidate reaction set that are considered for addition and y_j is a binary variable corresponding to the reactions *j* from the set *P*, whose values indicate addition (1) or no addition (0), we seek to the number of additions:

$$\min \sum_{j \in P} y_j \ , \tag{1}$$

subject to the fluxes v_j in the set of reactions associated with the FP gene set to zero, i.e.:

$$\text{s.t.} \qquad v_j = 0 \qquad \forall j \in \text{set of reactions associated with the FP gene,} \tag{2}$$

and the mass balance requirements summarized in *Equations 3-7*:

$$\sum_j S_{ij} v_j = 0, \qquad i = 1 \ldots M \tag{3}$$

$$\forall j \in \text{set of reactions in the network and } P$$

$$v_{biomass} \geq v_{biomass}^{cut-off} \tag{4}$$

$$LB_j \leq v_j \leq UB_j \qquad \forall j \in \text{set of reactions in the network} \tag{5}$$

$$LB_j y_j \leq v_j \leq UB_j y_j \qquad \forall j \in \text{set of reactions in } P \tag{6}$$

$$y_j = \{0,1\} \qquad \forall j \in \text{set of reactions in } P \tag{7}$$

where *j* represents the reaction indices, v_j indicates the flux for reaction *j*, *i* represents the metabolite indices, S_{ij} denotes the stoichiometric matrix, and *M* indicates the number of metabolites. *LB* and *UB* represent the lower bound and upper bound, respectively, and are taken directly from the *iNJ*661m network.

H. Optimization scheme 2: FN corrections

This optimization scheme attempts to find modification(s) associated with suppressing a minimum number of reactions that can correct a FN gene prediction. The calculations are done at two levels: an outer level, where the state of the network is changed, in this case by blocking and unblocking a set of reactions; and an inner level, which maximizes the growth rate using the FBA of that particular state of the metabolic network. If the resultant growth rate is below the minimum growth threshold, the FN prediction is corrected.

For a FN prediction, we want to change the network in such a way as to reduce the biomass accumulation rate (or growth rate) $v_{biomass}$ below a threshold value subject to the suppression of a number of candidate reactions from a given set *P*. The growth rate of the resultant network with the specified reactions suppressed is maximized using FBA, and network modifications that create accumulation rates less than a cutoff $v_{biomass} < v_{biomass}^{cut-off}$ are proposed to fix the FN prediction. We initially solved the model when the number of removed reactions n^* was 1. If the resultant biomass growth was above the minimum growth limit, $v_{biomass} \geq v_{biomass}^{cut-off}$, we increased n^* until $v_{biomass} < v_{biomass}^{cut-off}$.

This is summarized in *Equations* (*1-8*) , where *P* represents the set of candidate reactions that are to be suppressed and y_j is a binary variable corresponding to the reactions *j* in *P*, whose values indicate either suppression (0) or no suppression (1). The fluxes of reactions associated with FN gene set to zero (*Equation 6*). Given a particular set of values for y_j the inner optimization procedure (denoted as “[Inner]” below with its associated *Equations* (*3-5*) maximizes the biomass accumulation rate $v_{biomass}$ using FBA. The procedure minimizes the results of the inner optimization model by systematically changing the values for y_j, i.e., for a different set of suppressed reactions from *P*.

$$\min v_{biomass} \qquad (1)$$

$$\text{s.t.} \quad \max v_{biomass} \quad \text{[Inner]} \qquad (2)$$

$$\begin{bmatrix} \sum_j S_{ij} v_j = 0 \quad i = 1 \dots M & & (3) \\ LB_j \leq v_j \leq UB_j & \forall j \notin P & (4) \\ LB_j y_j \leq v_j \leq UB_j y_j & \forall j \in P & (5) \end{bmatrix}$$

$$v_j = 0 \qquad \forall j \in \text{set of reactions associated with the FN gene} \qquad (6)$$

$$\sum_j (1 - y_j) \leq n^* \qquad \forall j \in \text{set of reactions in } P \qquad (7)$$

$$y_j = \{0,1\} \qquad \forall j \in \text{set of reactions in } P \qquad (8)$$

For the above equations, *j* represents the reaction indices, v_j indicates the flux for reaction *j*, *i* represents the metabolite indices, S_{ij} denotes the stoichiometric matrix, and *M* indicates the number of metabolites. *LB* and *UB* represent lower bound and upper bound, respectively, and are taken directly from the *iNJ*661m network.

S3. Supplemental Tables and Figure

Supplemental Table S1. Summary of modifications to correct each gene essentiality prediction after *Step II*.

Of the 25 genes that were incorrectly predicted to be non-essential [false negative (FN)] under *in vivo* conditions, 18 genes were corrected and became essential after *Step II*. Of the 76 genes that were incorrectly predicted to be essential [false positive (FP)] under *in vivo* conditions, 24 genes were corrected and became non-essential after *Step II*. We classified the overall 42 (18 + 24) genes into 35 gene groups, defined as a group of genes whose products catalyze the same reaction(s).

Gene Group No.	Gene No.	Gene Locus	Gene Name	FP/FN	Pathway	Function/Reaction	Modification to Correct the False Gene Essentiality Prediction
1	1	*Rv1099c*	*Rv1099c*	FN	Glycolysis/ gluconeogenesis	Convert fructose-1,6-bisphosphate into fructose-6-phosphate	(1) Blocked the uptake of glucose from the environment
2	2	*Rv2702*	*ppgK*	FN	Glycolysis/ gluconeogenesis	Conversion between glucose-6-phosphate and glucose	(1) Blocked the function of the enzymes of fructose-bisphosphate aldolase and hexokinase<br>(2) Blocked the function of the enzymes of fructose-bisphosphatase and hexokinase<br>(3) Blocked the conversion from maltose to glucose and blocked the uptake of glucose<br>(4) Blocked the conversion between maltose and trehalose and blocked the uptake of glucose<br>(5) Blocked the function of the enzymes of glucose-6-phosphate isomerase and hexokinase
3	3<br>4<br>5	*Rv1350*<br>*Rv1483*<br>*Rv2947c*	*fabG2*<br>*inhA*<br>*pks15*	FP<br>FP<br>FP	Fatty acid metabolism	Synthesis of fatty acids	(1) Allowed the uptakes of the following fatty acids: hexadecanoate, octadecanoate, octanoate, dodecanoate, arachidic acid, and hexacosanoate
4	6	*Rv2503c*	*scoB*	FP	Fatty acid metabolism	Functions as 3-oxoacid CoA-transferase	(1) Let the reaction catalyzed by acetyl-CoA:acetoacetyl-CoA transferase be reversible
5	7	*Rv3229c*	*desA3*	FN	Fatty acid metabolism	Palmitoyl-CoA desaturation	(1) Blocked the synthesis of hexadecenoate

6	8	*Rv1185c*	*fadD21*	FN	Fatty acid metabolism	Synthesis of fatty acid-CoA	(1) Blocked the ability of *fadD9* (*Rv2590*), *fadD24* (*Rv1529*), and *fadD23* (*Rv3826*) to catalyze the synthesis of fatty acid-CoA
7	9	*Rv0098*	*Rv0098*	FN	Fatty acid metabolism	Mycolic acid synthesis	(1) Blocked the ability of *fabG1* (*Rv1483*) to catalyze the same reaction
8	10	*Rv2483c*	*plsC*	FN	Fatty acid metabolism	Synthesis of 1,2-diacyl-*sn*-glycerol 3-phosphate (a phospholipid)	(1) Blocked the ability of *Rv2182c* to catalyze the same reaction
9	11	*Rv1416*	*ribH*	FP	Vitamin and cofactor metabolism	Synthesis of riboflavin	(1) Removed riboflavin and FMN from the biomass objective function
10	12	*Rv1412*	*ribC*	FP	Vitamin and cofactor metabolism	Synthesis of riboflavin	(1) Removed riboflavin and FMN from the biomass objective function
11	13	*Rv2671*	*ribD*	FP	Vitamin and cofactor metabolism	Synthesis of a riboflavin precursor	(1) Removed riboflavin and FMN from the biomass objective function
12	14	*Rv2786c*	*ribF*	FP	Vitamin and cofactor metabolism	Synthesis of flavin mononucleotide (FMN) from riboflavin	(1) Removed FMN from the biomass objective function
13	15	*Rv2421c*	*Rv2421c*	FP	Vitamin and cofactor metabolism	Synthesis of deamino-NAD^+	(1) Removed NAD and NADPfrom the biomass objective function
14	16	*Rv1596*	*nadC*	FP	Vitamin and cofactor metabolism	Functions as nicotinate-nucleotide diphosphorylase	(1) Removed NADand NADPfrom the biomass objective function
15	17	*Rv3215*	*entC*	FP	Vitamin and cofactor metabolism	Synthesis of isochorismate	(1) Removed menaquinol 8 from the biomass objective fucntion
16	18	*Rv1568*	*bioA*	FN	Vitamin and cofactor metabolism	Synthesis of a precursor of biotin	(1) Added biotinyl-5'-AMP to the biomass objective function
17	19	*Rv1569*	*bioF*	FN	Vitamin and cofactor metabolism	Synthesis of a precursor of biotin	(1) Added biotinyl-5'-AMP to the biomass objective function and blocked ability of *bioF2* (*Rv0032*) to catalyze the same reaction
18	20	*Rv1589*	*bioB*	FN	Vitamin and cofactor metabolism	Synthesis of biotin	(1) Added biotinyl-5'-AMP to the biomass objective function
19	21	*Rv2211c*	*gcvT*	FN	Vitamin and cofactor metabolism	Conversion between 5-formyltetrahydrofolate and 5,10-methenyltetrahydrofolate	(1) Added the metabolite 5-formyltetrahydrofolate to the biomass objective function
20	22	*Rv3001c*	*ilvC*	FP	Amino acid metabolism	Synthesis of 2,3-dihydroxy-3-methylbutanoate and 2,3-dihydroxy-3-methylpentanoate	(1) Allowed the uptakes of isoleucine and valine

21	23	*Rv3002c*	*ilvN*	FP	Amino acid metabolism	Synthesis of acetolactate	(1) Allowed the uptake of valine
22	24<br>25<br>26<br>27	*Rv2220c*<br>*Rv1878*<br>*Rv2222c*<br>*Rv2860c*	*glnA1*<br>*glnA3*<br>*glnA2*<br>*glnA4*	FP<br>FP<br>FP<br>FP	Amino acid metabolism	Synthesis of glutamine	(1) Let the reaction of glutamate synthesis from glutamine be reversible<br>(2) Let the conversion to CTP and glutamate from UTP and glutamine be reversible
23	28	*Rv3754*	*tyrA*	FP	Amino acid metabolism	Functions as prephenate dehydrogenase	(1) Allowed the uptake of tyrosine
24	29	*Rv3042c*	*serB2*	FN	Amino acid metabolism	Remove a phosphate group from phosphoserine to produce serine	(1) Blocked the ability of *serB* (*Rv0505c*) to catalyze the same reaction
25	30	*Rv2231c*	*cobC*	FN	Amino acid metabolism	Convert glutamate into histidinol-phosphate	(1) Blocked the ability of *hisC2* (*Rv3772*) and *hisC* (*Rv1600*) to catalyze the conversion
26	31	*Rv2945c*	*lppX*	FN	Transport	Transport phthiocerol dimycocerosate A and phenol phthiocerol dimycocerosate out of the cell	(1) Added extracellular phthiocerol dimycocerosate A to the biomass objective function<br>(2) Added extracellular phenol phthiocerol dimycocerosate to the biomass objective function
27	32<br>33<br>34	*Rv1236*<br>*Rv1237*<br>*Rv1238*	*sugA*<br>*sugB*<br>*sugC*	FN	Transport	Transport of glucose, maltoheptaose, maltose, ribose, trehalose, and xylose into the cell	(1) Blocked the function of the enzyme of fructose-bisphosphate aldolase<br>(2) Blocked the function of the enzyme of fructose-bisphosphatase<br>(3) Blocked the function of the enzyme of glucose-6-phosphate isomerase<br>(4) Allowed the ribose uptake and blocked the inosine hydrolysis<br>(5) Allowed the xylose uptake,and added xylose to the biomass objective function
28	35	*Rv3236c*	*kefB*	FN	Transport	Transport of K^+ and Na^+ into the cell	(1) Blocked the function of potassium ABC transporter<br>(2) Blocked the function of the Na^+ antiporter
29	36	*Rv1699*	*pyrG*	FP	Nucleotide metabolism	Synthesis of CTP from UTP	(1) Allowed the uptake of cytidine
30	37	*Rv1385*	*pyrF*	FP	Nucleotide metabolism	Functions as orotidine-5'-phosphate decarboxylase	
31	38	*Rv2139*	*pyrD*	FP	Nucleotide metabolism	Functions as dihydroorotic acid dehydrogenase	

32	39	*Rv3393*	*iunH*	FP	Nucleotide metabolism	Hydrolysis of inosine	(1) Let the reaction catalyzed by ribokinase be reversible
33	40	*Rv2465c*	*rpi*	FP	Pentose phosphate pathway	Functions as ribose-5-phosphate isomerase	(1) Allowed the secretion of D-arabinose
34	41	*Rv3628*	*ppa*	FP	Multiple pathways	Functions as inorganic diphosphatase	(1) Let the reaction catalyzed by nucleoside triphosphate tripolyhydrolase of deoxy-GTP (dGTP) be reversible
35	42	*Rv3588c*	*Rv3588c*	FN	Multiple pathways	Conversion between carboxylic acid and carbon dioxide	(1) Blocked the ability of *Rv3273* to catalyze the same reaction

Supplemental Table S2. Synthetic essential gene pair predictions based on *iNJ*661m and *iNJ*661v.
Some gene pairs highlighted in color are those involving carbon metabolism (see Supplemental Fig. S1). The different colors refer to the underlying "reason" as to why the gene pairs were found to be essential in *iNJ*661v. Red gene pairs were located in glucose synthesis pathways, blue gene pairs were involved in the synthesis of 3-phospho-glycerate (3pg), and orange gene pairs contained two genes whose gene product catalyzed the same functions. In addition, green gene pairs contained at least one gene related to energy metabolism.

Gene Pairs Only Predicted to Be Essential Using *iNJ*661v					
Pair Number	*Gene 1*		*Gene 2*		Annotations
	Locus	Name	Locus	Name	
1	*Rv3696c*	*glpK*	*Rv1436*	*gap*	The gene *glpK* was necessary for the synthesis of glucose from glycerol.<br>The genes *gap*, *pgk*, *gpm*, *eno*, *fum*, and *glcB* were necessary for the synthesis of glucose from fatty acids.<br>The gene *tpi* was required in each pathway.
2	*Rv3696c*	*glpK*	*Rv1437*	*pgk*	
3	*Rv3696c*	*glpK*	*Rv0489*	*gpm*	
4	*Rv3696c*	*glpK*	*Rv1023*	*eno*	
5	*Rv3696c*	*glpK*	*Rv1098c*	*fum*	
6	*Rv3696c*	*glpK*	*Rv1438*	*tpi*	
7	*Rv3696c*	*glpK*	*Rv1837c*	*glcB*	
8	*Rv1438*	*tpi*	*Rv1436*	*gap*	
9	*Rv1438*	*tpi*	*Rv1437*	*pgk*	
10	*Rv1438*	*tpi*	*Rv0489*	*gpm*	
11	*Rv1438*	*tpi*	*Rv1023*	*eno*	
12	*Rv1438*	*tpi*	*Rv1098c*	*fum*	
13	*Rv1438*	*tpi*	*Rv1837c*	*glcB*	

14	*Rv1837c*	*glcB*	*Rv1436*	*gap*	The genes *fum* and *glcB* were necessary for the synthesis of 3pg, a precursor for serine synthesis, from fatty acids. The genes *gap* and *pgk* were necessary for the synthesis of 3pg from glycerol.
15	*Rv1837c*	*glcB*	*Rv1437*	*pgk*	
16	*Rv1098c*	*fum*	*Rv1436*	*gap*	
17	*Rv1098c*	*fum*	*Rv1437*	*pgk*	
18	*Rv0066c*	*icd2*	*Rv3339c*	*icd1*	The products of both genes catalyzed the conversion from isocitrate to α-ketoglutarate.
19	*Rv2476c*	*Rv2476c*	*Rv3858c*	*gltD*	*Rv2476c* was necessary for a reaction converting α-ketoglutarate into glutamate. The genes *gltB* and *gltD* were necessary for another reaction converting α-ketoglutarate into glutamate.
20	*Rv2476c*	*Rv2476c*	*Rv3859c*	*gltB*	
21	*Rv0468*	*fadB2*	*Rv1715*	*fadB3*	Either *fadB2* or *fadB3* was necessary for the β-oxidation of fatty acids.
22	*Rv1552*	*frdA*	*Rv3316*	*sdhC*	The products of both *frdA-frdD* and *sdhA-sdhD* catalyzed the conversion between succinate and fumarate. Alternative genes, *Rv0247c* and *Rv0248c*, could play the same role as *sdhB*; hence, it was not included in the listed essential gene pairs.
23	*Rv1553*	*frdB*	*Rv3316*	*sdhC*	
24	*Rv1554*	*frdC*	*Rv3316*	*sdhC*	
25	*Rv1555*	*frdD*	*Rv3316*	*sdhC*	
26	*Rv1552*	*frdA*	*Rv3317*	*sdhD*	
27	*Rv1553*	*frdB*	*Rv3317*	*sdhD*	
28	*Rv1554*	*frdC*	*Rv3317*	*sdhD*	
29	*Rv1555*	*frdD*	*Rv3317*	*sdhD*	
30	*Rv1552*	*frdA*	*Rv3318*	*sdhA*	
31	*Rv1553*	*frdB*	*Rv3318*	*sdhA*	
32	*Rv1554*	*frdC*	*Rv3318*	*sdhA*	
33	*Rv1555*	*frdD*	*Rv3318*	*sdhA*	

34	*Rv1161*	*narG*	*Rv1620c*	*cydC*	The genes *narG-narJ* and *narK2* were necessary components of the electron transport chain when nitrate was used to oxidize reduced NADH and FADH2. The genes *cydB-cydD* and *appC* were necessary for the electron transport chain when O_2 was used to oxidize NADH and FADH2.
35	*Rv1162*	*narH*	*Rv1620c*	*cydC*	
36	*Rv1163*	*narJ*	*Rv1620c*	*cydC*	
37	*Rv1164*	*narI*	*Rv1620c*	*cydC*	
38	*Rv1737c*	*narK2*	*Rv1620c*	*cydC*	
39	*Rv1161*	*narG*	*Rv1621c*	*cydD*	
40	*Rv1162*	*narH*	*Rv1621c*	*cydD*	
41	*Rv1163*	*narJ*	*Rv1621c*	*cydD*	
42	*Rv1164*	*narI*	*Rv1621c*	*cydD*	
43	*Rv1737c*	*narK2*	*Rv1621c*	*cydD*	
44	*Rv1161*	*narG*	*Rv1622c*	*cydB*	
45	*Rv1162*	*narH*	*Rv1622c*	*cydB*	
46	*Rv1163*	*narJ*	*Rv1622c*	*cydB*	
47	*Rv1164*	*narI*	*Rv1622c*	*cydB*	
48	*Rv1737c*	*narK2*	*Rv1622c*	*cydB*	
49	*Rv1161*	*narG*	*Rv1623c*	*appC*	
50	*Rv1162*	*narH*	*Rv1623c*	*appC*	
51	*Rv1163*	*narJ*	*Rv1623c*	*appC*	
52	*Rv1164*	*narI*	*Rv1623c*	*appC*	
53	*Rv1737c*	*narK2*	*Rv1623c*	*appC*	
54	*Rv1161*	*narG*	*Rv1304*	*atpB*	The genes *narG-narJ* and *narK2* were necessary components of the electron transport chain when nitrate was used to oxidize reduced NADH and FADH2. The gene products of *atpA-atpH* catalyzed synthesis of ATP.
55	*Rv1161*	*narG*	*Rv1305*	*atpE*	
56	*Rv1161*	*narG*	*Rv1306*	*atpF*	
57	*Rv1161*	*narG*	*Rv1307*	*atpH*	
58	*Rv1161*	*narG*	*Rv1308*	*atpA*	

59	*Rv1161*	*narG*	*Rv1309*	*atpG*	The genes *narG-narJ* and *narK2* were necessary components of the electron transport chain when nitrate was used to oxidize reduced NADH and FADH2.<br>The gene products of *atpA-atpH* catalyzed synthesis of ATP.
60	*Rv1161*	*narG*	*Rv1310*	*atpD*	
61	*Rv1161*	*narG*	*Rv1311*	*atpC*	
62	*Rv1162*	*narH*	*Rv1304*	*atpB*	
63	*Rv1162*	*narH*	*Rv1305*	*atpE*	
64	*Rv1162*	*narH*	*Rv1306*	*atpF*	
65	*Rv1162*	*narH*	*Rv1307*	*atpH*	
66	*Rv1162*	*narH*	*Rv1308*	*atpA*	
67	*Rv1162*	*narH*	*Rv1309*	*atpG*	
68	*Rv1162*	*narH*	*Rv1310*	*atpD*	
69	*Rv1162*	*narH*	*Rv1311*	*atpC*	
70	*Rv1163*	*narJ*	*Rv1304*	*atpB*	
71	*Rv1163*	*narJ*	*Rv1305*	*atpE*	
72	*Rv1163*	*narJ*	*Rv1306*	*atpF*	
73	*Rv1163*	*narJ*	*Rv1307*	*atpH*	
74	*Rv1163*	*narJ*	*Rv1308*	*atpA*	
75	*Rv1163*	*narJ*	*Rv1309*	*atpG*	
76	*Rv1163*	*narJ*	*Rv1310*	*atpD*	
77	*Rv1163*	*narJ*	*Rv1311*	*atpC*	
78	*Rv1164*	*narI*	*Rv1304*	*atpB*	
79	*Rv1164*	*narI*	*Rv1305*	*atpE*	
80	*Rv1164*	*narI*	*Rv1306*	*atpF*	
81	*Rv1164*	*narI*	*Rv1307*	*atpH*	
82	*Rv1164*	*narI*	*Rv1308*	*atpA*	
83	*Rv1164*	*narI*	*Rv1309*	*atpG*	
84	*Rv1164*	*narI*	*Rv1310*	*atpD*	

85	Rv1164	narI	Rv1311	atpC	The genes *narG-narJ* and *narK2* were necessary components of the electron transport chain when nitrate was used to oxidize reduced NADH and FADH2. The gene products of *atpA-atpH* catalyzed synthesis of ATP.
86	Rv1737c	narK2	Rv1304	atpB	
87	Rv1737c	narK2	Rv1305	atpE	
88	Rv1737c	narK2	Rv1306	atpF	
89	Rv1737c	narK2	Rv1307	atpH	
90	Rv1737c	narK2	Rv1308	atpA	
91	Rv1737c	narK2	Rv1309	atpG	
92	Rv1737c	narK2	Rv1310	atpD	
93	Rv1737c	narK2	Rv1311	atpC	
94	Rv0191	Rv0191	Rv1304	atpB	The gene products of *atpA-atpH* catalyzed synthesis of ATP. The gene product of *Rv0191* transported lactate out of the cell. The gene product of *nanT* transported lactate and pyruvate into or out of the cell. We noted that each of the gene pairs (94-109) involved one gene in *atpA-atpH* (ATP synthesis). Therefore, we considered these gene pairs to be related to energy metabolism.
95	Rv0191	Rv0191	Rv1305	atpE	
96	Rv0191	Rv0191	Rv1306	atpF	
97	Rv0191	Rv0191	Rv1307	atpH	
98	Rv0191	Rv0191	Rv1308	atpA	
99	Rv0191	Rv0191	Rv1309	atpG	
100	Rv0191	Rv0191	Rv1310	atpD	
101	Rv0191	Rv0191	Rv1311	atpC	
102	Rv1902c	nanT	Rv1304	atpB	
103	Rv1902c	nanT	Rv1305	atpE	
104	Rv1902c	nanT	Rv1306	atpF	
105	Rv1902c	nanT	Rv1307	atpH	
106	Rv1902c	nanT	Rv1308	atpA	
107	Rv1902c	nanT	Rv1309	atpG	
108	Rv1902c	nanT	Rv1310	atpD	
109	Rv1902c	nanT	Rv1311	atpC	

110	*Rv2920c*	*amt*	*Rv2391*	*nirA*	The gene product of *amt* transported NH_4^+ into the cell.<br>The gene products of *nirA*, *nirB*, *nirD*, *narG-narJ*, and *narK2* were necessary for the synthesis of NH_4^+.
111	*Rv2920c*	*amt*	*Rv0252*	*nirB*	
112	*Rv2920c*	*amt*	*Rv0253*	*nirD*	
113	*Rv2920c*	*amt*	*Rv1161*	*narG*	
114	*Rv2920c*	*amt*	*Rv1162*	*narH*	
115	*Rv2920c*	*amt*	*Rv1163*	*narJ*	
116	*Rv2920c*	*amt*	*Rv1164*	*narI*	
117	*Rv2920c*	*amt*	*Rv1737c*	*narK2*	
118	*Rv1699*	*pyrG*	*Rv1712*	*cmk*	The gene product of *pyrG* was necessary for the synthesis of CTP from UTP.<br>The gene product of *cmk* was necessary for the synthesis of CTP from cytidine.
119	*Rv1098c*	*fum*	*Rv2465c*	*rpi*	*fum* encoded fumarase, and was part of citric acid cycle.<br>*rpi* encoded ribose-5-phosphate isomerise.
120	*Rv2465c*	*rpi*	*Rv1408*	*rpe*	*rpe*, *tal*, and *rpi* encoded ribulose 5-phosphate 3-epimerase, transaldolase, and ribose-5-phosphate isomerise, respectively. The three genes were part of pentose phosphate pathway.<br>*glcB* encoded malate synthase, and was part of glyoxylate cycle.
121	*Rv2465c*	*rpi*	*Rv1448c*	*tal*	
122	*Rv2465c*	*rpi*	*Rv1837c*	*glcB*	
123	*Rv2436*	*rbsK*	*Rv3393*	*iunH*	*rbsK* and *iunH* encoded ribokinase and inosine hydrolase, respectively, and were part of nucleotide metabolism.
124	*Rv2344c*	*dgt*	*Rv3628*	*ppa*	*dgt* encoded nucleoside triphosphate tripolyhydrolase. *ppa* encoded inorganic diphosphatase.
125	*Rv0533c*	*fabH*	*Rv1350*	*fabG2*	*fabH*, *fabG2*, *inhA*, *fas*, and *pks15* encoded enzymes for the synthesis of tetradecanoate.
126	*Rv0533c*	*fabH*	*Rv1484*	*inhA*	
127	*Rv0533c*	*fabH*	*Rv2524c*	*fas*	
128	*Rv0533c*	*fabH*	*Rv2947c*	*pks15*	

129	*Rv2139*	*pyrD*	*Rv3316*	*sdhC*	*pyrD* encoded dihydroorotic acid dehydrogenase, and was part of pyrimidine metabolism. *sdhA*, *sdhC*, and *sdhD* encoded succinate dehydrogenase in citric acid cycle.
130	*Rv2139*	*pyrD*	*Rv3317*	*sdhD*	
131	*Rv2139*	*pyrD*	*Rv3318*	*sdhA*	
Gene Pairs Only Predicted to Be Essential Using *iNJ*661m					
Pair Number	*Gene 1*		*Gene 2*		Annotations
	Locus	Name	Locus	Name	
1	*Rv1236*	*sugA*	*Rv0946c*	*pgi*	The genes *sugA*-*sugC* were necessary for glucose uptake.<br>The genes *pgi*, *Rv1099c*, and *fba* were necessary for glucose synthesis.
2	*Rv1237*	*sugB*	*Rv0946c*	*pgi*	
3	*Rv1238*	*sugC*	*Rv0946c*	*pgi*	
4	*Rv1236*	*sugA*	*Rv1099c*	*Rv1099c*	
5	*Rv1237*	*sugB*	*Rv1099c*	*Rv1099c*	
6	*Rv1238*	*sugC*	*Rv1099c*	*Rv1099c*	
7	*Rv1236*	*sugA*	*Rv0363c*	*fba*	
8	*Rv1237*	*sugB*	*Rv0363c*	*fba*	
9	*Rv1238*	*sugC*	*Rv0363c*	*fba*	
10	*Rv1436*	*gap*	*Rv1304*	*atpB*	The genes *gap*, *pgk*, and *tpi* were in the glycolysis pathway that synthesized ATP.<br>The gene products of *atpA*-*atpH* synthesized ATP from ADP and extracellular H^+.
11	*Rv1437*	*pgk*	*Rv1304*	*atpB*	
12	*Rv1438*	*tpi*	*Rv1304*	*atpB*	
13	*Rv1436*	*gap*	*Rv1305*	*atpE*	
14	*Rv1437*	*pgk*	*Rv1305*	*atpE*	
15	*Rv1438*	*tpi*	*Rv1305*	*atpE*	

16	Rv1436	gap	Rv1306	atpF	The genes *gap*, *pgk*, and *tpi* were in the glycolysis pathway that synthesized ATP. The gene products of *atpA*-*atpH* synthesized ATP from ADP and extracellular H^+.
17	Rv1437	pgk	Rv1306	atpF	
18	Rv1438	tpi	Rv1306	atpF	
19	Rv1436	gap	Rv1307	atpH	
20	Rv1437	pgk	Rv1307	atpH	
21	Rv1438	tpi	Rv1307	atpH	
22	Rv1436	gap	Rv1308	atpA	The genes *gap*, *pgk*, and *tpi* were in the glycolysis pathway that synthesized ATP. The gene products of *atpA*-*atpH* synthesized ATP from ADP and extracellular H^+.
23	Rv1437	pgk	Rv1308	atpA	
24	Rv1438	tpi	Rv1308	atpA	
25	Rv1436	gap	Rv1309	atpG	
26	Rv1437	pgk	Rv1309	atpG	
27	Rv1438	tpi	Rv1309	atpG	
28	Rv1436	gap	Rv1310	atpD	
29	Rv1437	pgk	Rv1310	atpD	
30	Rv1438	tpi	Rv1310	atpD	
31	Rv1436	gap	Rv1311	atpC	
32	Rv1437	pgk	Rv1311	atpC	
33	Rv1438	tpi	Rv1311	atpC	
34	Rv3236c	kefB	Rv1029	kdpA	The gene product of *kefB* transported potassium into the cell. The gene products of *kdpA*-*kdpC* also transported potassium into the cell.
35	Rv3236c	kefB	Rv1030	kdpB	
36	Rv3236c	kefB	Rv1031	kdpC	
37	Rv3236c	kefB	Rv2287	yjcE	The gene product of *kefB* transported sodium into the cell. The gene product of *yjcE* also transported sodium into the cell.
38	Rv3042	serB2	Rv0505c	serB	The gene products of both *serB* and *serB2* converted phosphoserine into serine.
39	Rv2483c	plsC	Rv2182c	Rv2182c	The products of both genes catalyzed the synthesis of 1,2-diacyl-*sn*-glycerol 3-phosphate (a phospholipid).

40	*Rv1483*	*fabG1*	*Rv0098*	*Rv0098*	The products of both genes catalyzed the synthesis of mycolic acid.
41	*Rv3588c*	*Rv3588c*	*Rv3273*	*Rv3273*	Two enzymes from these two genes catalyzed the synthesis or degradation of H_2CO_3.
42	*Rv3307*	*deoD*	*Rv3624c*	*hpt*	The genes *deoD* and *hpt* encoded purine-nucleoside phosphorylase and hypoxanthine phosphoribosyltransferase, respectively, during purine metabolism.
43	*Rv3356c*	*folD*	*Rv2945c*	*lppX*	Available biological and metabolic information is incomplete.
Gene Pairs Predicted to Be Essential in both *iNJ*661m and *iNJ*661v					
Pair Number	*Gene 1*		*Gene 2*		Annotations
	Locus	Name	Locus	Name	
1	*Rv0070c*	*glyA2*	*Rv1093*	*glyA*	Both gene products functioned as glycine hydroxymethyltransferases during amino acid metabolism.
2	*Rv1295*	*thrC*	*Rv1559*	*ilvA*	The genes *thrC*, *thrB*, and *ilvA* encoded threonine synthase, homoserine kinase, and threonine deaminase, respectively, and were part of different amino acid metabolism pathways.
3	*Rv1296*	*thrB*	*Rv1559*	*ilvA*	
4	*Rv1001*	*arcA*	*Rv1658*	*argG*	The genes *arcA*, *argG*, and *argH* encoded arginine deiminase, argininosuccinate synthase, and argininosuccinate lyase, respectively, and were part of different amino acid metabolism pathways.
5	*Rv1001*	*arcA*	*Rv1659*	*argH*	
6	*Rv2321c*	*rocD2*	*Rv2427c*	*proA*	The products of *rocD1* and *rocD2* functioned together as ornithine transaminase during amino acid metabolism.<br>The genes *proA* and *proB* encoded glutamate-5-semialdehyde dehydrogenase and glutamate-5-kinase, respectively, and were part of the urea cycle.
7	*Rv2322c*	*rocD1*	*Rv2427c*	*proA*	
8	*Rv2321c*	*rocD2*	*Rv2439c*	*proB*	
9	*Rv2322c*	*rocD1*	*Rv2439c*	*proB*	
10	*Rv1609*	*trpE*	*Rv2859c*	*Rv2859c*	Both gene products functioned as anthranilate synthases during amino acid metabolism.
11	*Rv0728c*	*Rv0728c*	*Rv2996c*	*serA*	Both gene products functioned as phosphoglycerate dehydrogenases during amino acid metabolism.
12	*Rv0337c*	*aspC*	*Rv3565*	*aspB*	Both gene products functioned as aspartate transaminases during amino acid metabolism.

13	*Rv0389*	*purT*	*Rv0956*	*purN*	Both gene products functioned as the enzyme of phosphoribosylglycinamide formyltransferases during purine metabolism.
14	*Rv0808*	*purF*	*Rv1602*	*hisH*	Both gene products functioned as glutamine phosphoribosyldiphosphate amidotransferases during purine metabolism.
15	*Rv2754c*	*Rv2754c*	*Rv2764c*	*thyA*	Both gene products functioned as thymidylate synthases during pyrimidine metabolism.
16	*Rv1712*	*cmk*	*Rv2883c*	*pyrH*	The gene *cmk* encoded cytidylate kinase and uridine monophosphate kinase during pyrimidine metabolism. The gene *pyrH* encoded uridylate kinase, which was active during nucleotide sugar metabolism.
17	*Rv0295c*	*Rv0295c*	*Rv1373*	*Rv1373*	Both gene products functioned as trehalose sulfotransferases during membrane metabolism.
18	*Rv0489*	*gpm*	*Rv1436*	*gap*	The genes *gpm* and *eno* encoded phosphoglycerate mutase and enolase, respectively, and were part of glycolysis/gluconeogenesis.<br>The genes *gap* and *pgk* encoded glyceraldehyde-3-phosphate dehydrogenase and phosphoglycerate kinase, respectively, were part of glycolysis/gluconeogenesis.
19	*Rv1023*	*eno*	*Rv1436*	*gap*	
20	*Rv0489*	*gpm*	*Rv1437*	*pgk*	
21	*Rv1023*	*eno*	*Rv1437*	*pgk*	
22	*Rv1408*	*rpe*	*Rv1445c*	*devB*	The genes *rpe*, *devB*, and *tal* encoded ribulose-5-phosphate 3-epimerase, 6-phosphogluconolactonase, and transaldolase, respectively, in the pentose phosphate pathway.
23	*Rv1408*	*rpe*	*Rv1448c*	*tal*	
24	*Rv0112*	*gca*	*Rv1511*	*gmdA*	Both gene products functioned as guanosine diphosphate-mannose dehydratases during sugar metabolism.
25	*Rv1739c*	*Rv1739c*	*Rv2397c*	*cysA*	The gene product of *Rv1739c* transported sulfate from environment.<br>The products of *cysA*, *cysW*, *cysT*, and *subI* functioned together as another sulfate transporter.
26	*Rv1739c*	*Rv1739c*	*Rv2398c*	*cysW*	
27	*Rv1739c*	*Rv1739c*	*Rv2399c*	*cysT*	
28	*Rv1739c*	*Rv1739c*	*Rv2400c*	*subI*	
29	*Rv1604*	*impA*	*Rv2701c*	*suhB*	Both gene products functioned as myo-inositol 1-phosphatases during fatty acid metabolism.
30	*Rv1822*	*pgsA2*	*Rv2746c*	*pgsA3*	Both gene products functioned as phosphatidylglycerol synthases during fatty acid metabolism and membrane metabolism.

31	*Rv0649*	*fadD37*	*Rv2243*	*fabD*	Both gene products functioned as malonyl-CoA-ACP transacylases during fatty acid metabolism.
32	*Rv1529*	*fadD24*	*Rv3826*	*fadD23*	Both gene products functioned as fatty-acid-CoA ligases during fatty acid metabolism.
33	*Rv0904c*	*accD3*	*Rv3281*	*accE5*	Both gene products were involved in the conversion between acetyl-CoA and malonyl-CoA during pyruvate metabolism.
34	*Rv2682c*	*dxs*	*Rv3379c*	*Rv3379c*	Both gene products functioned as 1-deoxy-xylulose 5-phosphate synthases during polyprenyl metabolism.
35	*Rv1207*	*folP2*	*Rv3608c*	*folP*	Both gene products functioned as dihydropteroate synthases during vitamin and cofactor metabolism.

Supplemental Table S3. Comparison of predicted gene essentiality using *iNJ*661v at different time points post-infection.
A true positive (TP) prediction refers to a gene correctly predicted to be essential, whereas a false negative (FN) prediction refers to a gene incorrectly predicted to be non-essential. A false positive (FP) prediction refers to a gene incorrectly predicted to be essential, whereas a true negative (TN) prediction refers to a gene correctly predicted to be non-essential. Sensitivity = TP/(TP + FN). Specificity = TN/(TN + FP). Matthews correlation coefficient = $(TP \times TN - FP \times FN)/[(TP + FP)(TP + FN)(TN + FP)(TN + FN)]^{½}$.

Network	Time Points Post-Infection (week)	Threshold	Number of Gene Essentiality Predictions				Sensitivity	Specificity	Matthews Correlation Coefficient
			TP	FN	FP	TN			
*iNJ*661v	1	≤0.2	13	2	67	293	0.87	0.81	0.33
	2		15	1	65	294	0.94	0.82	0.37
	4		17	3	63	292	0.85	0.82	0.37
	8		16	2	64	293	0.89	0.82	0.37

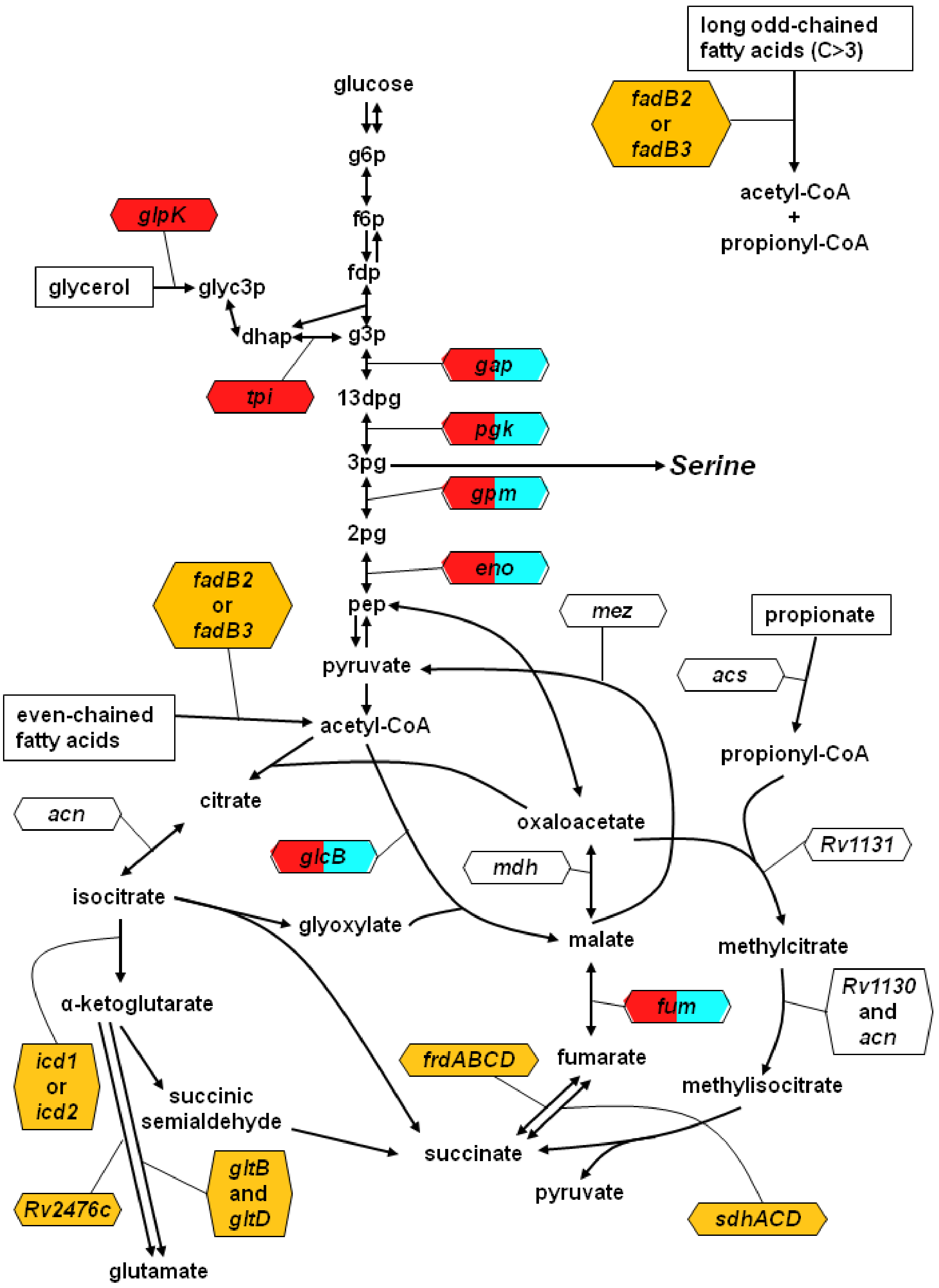

Supplemental Figure S1

Supplemental Figure S1. Mapping of synthetic essential gene pairs in *iNJ*661v to carbon metabolism-related pathways.

Arrows indicate metabolic reactions. Text without frames represents metabolites, text within a rectangular frame indicates material obtained from the host environment, and text within a hexagonal frame represents a gene. Genes marked with one or more colors belong to one or more predicted essential gene pairs. The colors indicate the underlying “reason” as to why the gene pairs were found to be essential in *iNJ*661v as shown in Supplemental Table 2.
